# DFT Investigation of 4-Mercaptoimidazole–Copper(I/II) Redox Chemistry and C2-Substituent Effects: A Minimal Scaffold Study of Ovothiol-Type Redox Cycling

**DOI:** 10.3390/molecules31183331

**Published:** 2026-09-19

**Authors:** Do Tuong Ha, Thuat T. Trinh

**Affiliations:** 1 Group of Applied Research in Advanced Materials for Sustainable Development, Faculty of Applied Sciences, Ton Duc Thang University, Ho Chi Minh City 70000, Vietnam; dotuongha@tdtu.edu.vn; 2 Porelab, Department of Chemistry and Biomedical Science, Norwegian University of Science and Technology, NO-7491 Trondheim, Norway

**Keywords:** DFT, 4-mercaptoimidazole, copper, reduction potential, ovothiol, redox cycling, IEFPCM, Hammett analysis, C2-substituent effects, electronic structure, QTAIM

## Abstract

4-Mercaptoimidazole (4MC) is the minimal parent scaffold of the ovothiol family of redox-active mercaptohistidines. We present a DFT study of the tautomerism, copper coordination, redox chemistry, and electronic structure of 4MC and its C2-substituted derivatives in aqueous solution. Twelve parent species (neutral tautomers, anions, disulfides, and Cu(I)/Cu(II) complexes) were optimised at BP86/def2-SVP with single-point energies at BP86/def2-TZVP/IEFPCM(water). The thione tautomer (*N3*-H) and its anion dominate ($\Delta G=-2.56\;\mathrm{kJ}\;{\mathrm{mol}}^{-1}$ and $-14.63\;\mathrm{kJ}\;{\mathrm{mol}}^{-1}$), leaving *N1* free for copper coordination; the *cis*-(*N3*) Cu(II) isomer is the global minimum (81%). The disulfide/thiol and Boltzmann-weighted Cu(II)/Cu(I) reduction potentials are $E^{\circ }=0.286\;V$ and 0.828 V vs. SHE, giving a positive EMF of 0.542 V comparable to the natural ovothiols. An M06-2X functional benchmark quantifies the method contribution, raising the copper potential to 1.063 V and confirming that the residual offset to the ovothiol literature values reflects the minimal scaffold. Three C2 derivatives (CH_3_, Cl, NO_2_; $\sigma_{p}$ from −0.17 to +0.78) show weak Hammett correlations for both redox couples, yet the EMF remains positive for all of them (0.302 to 0.738 V), indicating that C2 substitution tunes the thermodynamic coupling between the thiol–disulfide and copper centres without disrupting it. Electronic structure analysis (NBO, QTAIM) reveals polar covalent Cu–S bonding, spin delocalisation onto sulfur in Cu(II) complexes, and Cu–S dissociation with a switch to Cu–N coordination in the NO_2_ derivative. The core mercaptoimidazole scaffold thus retains the essential ovothiol redox chemistry, and C2 modification offers a genuine handle for modulating the driving force of the redox cycle.

## 1. Introduction

Ovothiols are a family of redox-active mercaptohistidine compounds originally isolated from marine invertebrate eggs by Turner and Shapiro [1]. These unique thiol-bearing histidine derivatives, namely ovothiol A, B, and C, differ in their *N1*-methylation pattern and have been identified in a wide range of marine organisms, from protozoa to invertebrates [2,3]. The biosynthesis of ovothiol A proceeds via the mercaptoimidazole core [3], making 4-mercaptoimidazole (4MC) the minimal structural parent of this family.

The biological significance of ovothiols stems from their potent antioxidant activity, which is intimately linked to their copper redox chemistry. Ovothiols belong to a broader family of bioactive thiols of microbial and marine origin [4] and have been specifically characterised as antioxidants in trypanosomatid parasites [5]. Copper is an essential trace element that cycles between the Cu(I) and Cu(II) oxidation states in numerous biological processes, including electron transfer, oxygen transport, and oxidative stress response [6,7,8]. Thiol–disulfide redox chemistry is equally central to cellular signalling and antioxidant defence [9]. The interplay between thiol/disulfide redox and copper redox cycling, wherein Cu(II) oxidises thiols to disulfides while being reduced to Cu(I), is a hallmark of ovothiol biochemistry and has motivated several computational studies.

De Luna et al. [10] reported the first DFT investigation of ergothioneine and ovothiol binding to copper, using the M06-2X/6-311+G(2df,p) level of theory with the SMD solvation model. Their study established the computational framework for calculating reduction potentials of the disulfide/thiol and Cu(II)/Cu(I) couples using the Llano and Eriksson [11] thermodynamic cycle. Wiebe et al. [12] extended this work to the selenium analogues of ergothioneine and ovothiol, demonstrating that chalcogen substitution modulates the copper reduction potential and overall redox cycling thermodynamics. DFT methods have also been employed to model the redox mechanisms of related thiol and selenol antioxidant systems [13].

Beyond ovothiol chemistry, DFT and ab initio molecular dynamics methods have proven broadly powerful for studying metal-ion interactions in aqueous solution [14,15] and reaction mechanisms in diverse chemical systems, including silicate condensation [16,17] and the stability of zeolite precursor complexes [18], and have recently been applied to radical scavenging in bioactive natural products [19].

These studies focused on the fully substituted ovothiol and ergothioneine structures, which bear an α-amino/α-carboxyl side chain and, in the case of ovothiols, a trimethylammonium group. It remains unclear to what extent the minimal mercaptoimidazole core retains the redox chemistry of its more elaborate natural derivatives. Resolving this is relevant not only for understanding the structure–activity relationship of ovothiols but also for the rational design of simplified antioxidant scaffolds [20,21,22].

In this study, we present a comprehensive DFT investigation of 4MC–copper redox chemistry. We examine the full tautomeric, protonation, redox, and isomeric (cis/trans, N1/N3-binding) landscape of the 4MC–copper system, encompassing neutral tautomers, anionic forms, disulfides, and Cu(I)/Cu(II) complexes. Reduction potentials of the disulfide/thiol and Cu(II)/Cu(I) couples are computed and related to the redox cycling underlying ovothiol antioxidant activity. To probe the substituent effect on the redox chemistry, a Hammett series of C2-substituted derivatives spanning electron-donating to strongly electron-withdrawing groups is analysed. A detailed electronic structure analysis further characterises the Cu–S bonding and the effect of substitution. The computational details are described in Section 2.

## 2. Materials and Methods

### 2.1. Level of Theory

All calculations were performed with Q-Chem 6.1.3 [23]. A two-step basis set protocol was employed for computational efficiency and accuracy. Geometry optimization and frequency analysis were performed using the BP86 [24,25] pure GGA functional with the def2-SVP split-valence basis set [26] and the RI-J approximation [27] with the corresponding rij-def2-SVP auxiliary basis [28]; all geometry optimizations were carried out in the gas phase, and analytical harmonic frequency analysis confirmed that all structures are true minima (zero imaginary frequencies). Single-point energies were then computed at the BP86/def2-TZVP [26] level with the IEFPCM [29] solvation model for water (ε=78.4). The PCM cavity was constructed using UFF radii with a van der Waals scale factor of 1.1, and the Lebedev grid was set to 110 points for both heavy atoms and hydrogen atoms. For five representative species (L2, L4, C2, D4, and NO_2_–Cu(II)), additional single-point calculations were performed for Quantum Theory of Atoms in Molecules (QTAIM) analysis using Multiwfn 3.8 [30] and Natural Bond Orbital (NBO) analysis [31], yielding Natural Population Analysis (NPA) charges and Wiberg bond indices; the NBO analysis was extended to the Cl-substituted copper complexes Cl-C2 and Cl-D4, and to the disulfide pair L6 and Cl-L6, to characterize the electronic-structure origin of the Cl outlier in the Hammett series (Section 3.8); for the two disulfides, the NBO analysis was performed without the PCM solvation model (a technical incompatibility for this system in Q-Chem 6.1.3), so that the parent-versus-Cl comparison is internally consistent at the gas-phase level. To assess the functional dependence of the computed redox potentials, the key parent species (L2, L6, C2, and the four Cu(II) isomers) were additionally evaluated by M06-2X [32]/def2-TZVP/IEFPCM single-point calculations on the same BP86/def2-SVP optimized geometries, with the same thermal corrections (Equation (1)); this benchmark quantifies the deviation introduced by the BP86 functional (Section 3.7).

The Gibbs free energy in solution was computed as:

(1) $$G_{\mathrm{soln}}^{\circ }=E_{\mathrm{SP}}^{\mathrm{PCM}}+\Delta G_{\mathrm{corr}}^{\mathrm{gas}}$$

where $E_{\mathrm{SP}}^{\mathrm{PCM}}$ is the single-point energy with IEFPCM solvation at the def2-TZVP level and $\Delta G_{\mathrm{corr}}^{\mathrm{gas}}$ is the gas-phase thermal correction (zero-point energy plus thermal and entropic contributions) obtained from the frequency analysis at the def2-SVP level, computed at 298.15 K and 1 atm following standard statistical mechanical expressions [33].

### 2.2. SCF Procedures

The default DIIS (direct inversion in the iterative subspace) SCF algorithm was used for most species. For the disulfides (L5, L6), Cu(I) complexes (C1, C2), and one Cu(II) isomer (D2), the geometric direct minimization (GDM) algorithm [34] was employed with a convergence threshold of $5\times 10^{-5}$. All single-point calculations used DIIS exclusively. Copper-containing complexes were optimized using Cartesian coordinates.

### 2.3. Species Studied

**Figure 1:**
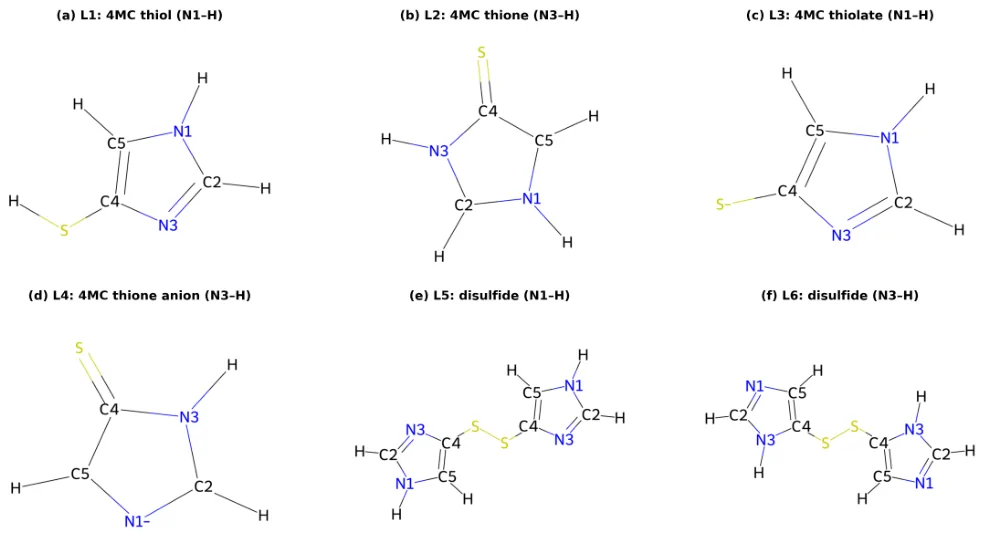
Chemical structures of the six 4-mercaptoimidazole ligand species investigated in this work.

Twenty-seven species were investigated: twelve parent 4MC species encompassing the full redox and protonation landscape, and fifteen C2-substituted derivatives (three substituents × five species each) for Hammett analysis (Table 1; Figure 1).

The 4MC molecule (C_3_H_4_N_2_S, MW = 100.14 g mol^−1^) consists of an imidazole ring with nitrogen atoms at positions *N1* and *N3*, and a sulfur atom at position C4. In the thiol tautomer, the hydrogen resides on *N1* and C4 bears a thiol (–SH) group; in the thione tautomer, the hydrogen migrates to *N3* and C4 adopts a thione (C=S) form. The Cu(I) complexes were modelled with three 4MC ligands in a monodentate S-coordination geometry, consistent with the tricoordinate preference of Cu(I) [10]. The Cu(II) complexes were modelled with two deprotonated 4MC^−^ ligands in a bidentate (S,N) chelation mode, with both *cis* and *trans* arrangements and both *N1* and *N3* binding motifs.

To probe substituent effects on the redox chemistry, three C2-substituted derivatives were studied at the same level of theory: CH_3_ ($\sigma_{p}=-0.17$), Cl ($\sigma_{p}=+0.23$), and NO_2_ ($\sigma_{p}=+0.78$). For each substituent, five species were computed, mirroring the dominant parent forms: the thione tautomer (L2 type), the N3-H thione anion (L4 type), the homodimeric disulfide (L6 type), the Cu(I) complex (C2 type), and the dominant D4 *cis*-*N3* Cu(II) isomer. This design provides a Hammett series spanning electron-donating to strongly electron-withdrawing substituents (Section 3.8).

### 2.4. Reduction Potential Calculations

Standard reduction potentials were computed using the thermodynamic cycle of Llano and Eriksson [11], who established the chemical potentials of the aqueous electron and proton relative to the SHE:

(2) $$\begin{array}{cc} \mu [e_{\mathrm{SHE}}^{-}\left(\mathrm{aq}\right)] & =-418.5\;\mathrm{kJ}\;{\mathrm{mol}}^{-1} \end{array}$$

(3) $$\begin{array}{cc} \mu [H^{+}\left(\mathrm{aq}\right)] & =-1124.2\;\mathrm{kJ}\;{\mathrm{mol}}^{-1} \end{array}$$

For the disulfide/thiol couple (Equation (1)), the reduction half-reaction is:

(4) $${\left(4\mathrm{MC}\right)}_{2}+2\;H^{+}\left(\mathrm{aq}\right)+2\;e_{\mathrm{SHE}}^{-}⟶2\;4{\mathrm{MC}}^{t}$$

where 4MC^t^ denotes the dominant tautomer (thione). The reaction free energy (products − reactants) and reduction potential are:

(5) $$\begin{array}{cc} \Delta_{r}G_{\mathrm{eq}1}^{\circ } & =2\;G^{\circ }\left[4{\mathrm{MC}}^{t}\right]-G^{\circ }\left[{\left(4\mathrm{MC}\right)}_{2}\right]-2\;\mu \left[H^{+}\right]-2\;\mu \left[e^{-}\right] \end{array}$$

(6) $$\begin{array}{cc} E_{\mathrm{eq}1}^{\circ } & =-\frac{\Delta_{r}G_{\mathrm{eq}1}^{\circ }}{2F} \end{array}$$

where $F=96485.33212\;C\;{\mathrm{mol}}^{-1}$ is the Faraday constant.

For the Cu(II)/Cu(I) complex couple (Equation (2)), the Cu(I) and Cu(II) complexes have different ligand stoichiometries (three neutral thione ligands vs. two monoanionic thiolate ligands), so the balanced reduction half-reaction couples both redox processes. Per two copper centres (n=2):

(7) $$2\;\mathrm{Cu}\left(\mathrm{II}\right){\left(4{\mathrm{MC}}^{-}\right)}_{2}+2\;4{\mathrm{MC}}^{t}+4\;H^{+}\left(\mathrm{aq}\right)+2\;e_{\mathrm{SHE}}^{-}⟶2\;\mathrm{Cu}\left(I\right){{\left(4{\mathrm{MC}}^{t}\right)}_{3}}^{+}$$

The reaction free energy and reduction potential are:

(8) $$\begin{array}{cc} \Delta_{r}G_{\mathrm{eq}2}^{\circ } & =2\;G^{\circ }\left[\mathrm{Cu}(I)\right]-\left\{2\;G^{\circ }\left[\mathrm{Cu}(\mathrm{II})\right]+2\;G^{\circ }\left[4{\mathrm{MC}}^{t}\right]+4\;\mu \left[H^{+}\right]+2\;\mu \left[e^{-}\right]\right\} \end{array}$$

(9) $$\begin{array}{cc} E_{\mathrm{eq}2}^{\circ } & =-\frac{\Delta_{r}G_{\mathrm{eq}2}^{\circ }}{2F} \end{array}$$

For the Cu(II)/Cu(I) couple, $E^{\circ }$ was computed for each of the four Cu(II) isomers (D1–D4) individually, and the Boltzmann-weighted average was reported as the final value:

(10) $$E_{\mathrm{avg}}^{\circ }=\sum_{i}P_{i}\;E_{i}^{\circ },\;P_{i}=\frac{\exp(-\Delta G_{i}/RT)}{\sum_{j}\exp\left(-\Delta G_{j}/RT\right)}$$

The overall electromotive force (EMF) for the coupled reaction (disulfide formation driven by Cu(II)-to-Cu(I) reduction) is:

(11) $$\mathrm{EMF}=E_{Cu(II)/Cu(I)}^{\circ }-E_{\mathrm{disulfide}/\mathrm{thiol}}^{\circ }$$

## 3. Results and Discussion

### 3.1. Tautomer Analysis

The two neutral tautomers of 4MC, thiol (*N1*-H, C4–SH; L1) and thione (*N3*-H, C4=S; L2), were optimized and their solution-phase Gibbs free energies computed (Table 2; Figure 1). The thione tautomer (L2) is lower in energy by

(12) $$\Delta G_{\mathrm{taut}}=G^{\circ }\left(\mathrm{thione}\right)-G^{\circ }\left(\mathrm{thiol}\right)=-2.56\;\mathrm{kJ}\;{\mathrm{mol}}^{-1}$$

confirming that the thione form is the dominant tautomer in aqueous solution. This result is consistent with the ovothiol literature, where thione tautomers are consistently found to be more stable in solution [10,12].

### 3.2. Anion Tautomer Preference

Upon deprotonation, two anionic forms are possible: the thiolate (*N1*-H, S^−^; L3) and the thione anion (*N3*-H, *N1*^−^; L4). The latter places the negative charge on the imidazole nitrogen *N1*, which is the copper-binding site in the ovothiol model [10]. Our calculations show that the thione anion (L4) is preferred by:

(13) $$\Delta G_{\mathrm{anion}}=G^{\circ }\left(N3\text{-}H\;\mathrm{anion}\right)-G^{\circ }\left(N1\text{-}H\;\mathrm{anion}\right)=-14.63\;\mathrm{kJ}\;{\mathrm{mol}}^{-1}$$

This 14.63 kJ mol^−1^ preference for the *N3*-H thione anion is significant: it confirms that deprotonation occurs at *N1* (not S), leaving the nitrogen lone pair available for copper coordination. The disulfide species (L5, L6) show a similarly small energy difference (ΔG<0.2 kJ mol^−1^), with the thione disulfide (L6) being marginally lower in energy, consistent with the thione tautomer dominance.

The preference for *N1* deprotonation over thiol formation has important mechanistic consequences. Because the anion retains the C=S thione character rather than adopting a thiolate (S^−^) form, copper binding occurs through the deprotonated *N1* nitrogen and the thione sulfur, consistent with the bidentate coordination mode observed in the ovothiol Cu(I) complexes of De Luna et al. [10]. In that study, the same *N3*-H thione anion was identified as the reactive species for copper chelation, and our 14.63 kJ mol^−1^ energy gap is consistent in both direction and magnitude with their reported values for ovothiol A (10 kJ mol^−1^ to 20 kJ mol^−1^ depending on the level of theory). This agreement indicates that the minimal 4MC scaffold reproduces the deprotonation selectivity of the full ovothiol system.

Furthermore, the persistence of thione dominance across the neutral (L2), anionic (L4), and disulfide (L6) species indicates a coherent thermodynamic thread throughout the redox cycle: the same tautomeric preference that governs the free ligand also stabilises the anion and the oxidised product. This consistency simplifies the mechanistic picture, as a single tautomeric form (thione) can be used to describe the entire Cu(II)-mediated thiol/disulfide cycle without invoking tautomerisation steps between redox events.

### 3.3. Cu(II) Isomer Energies and Populations

**Figure 2:**
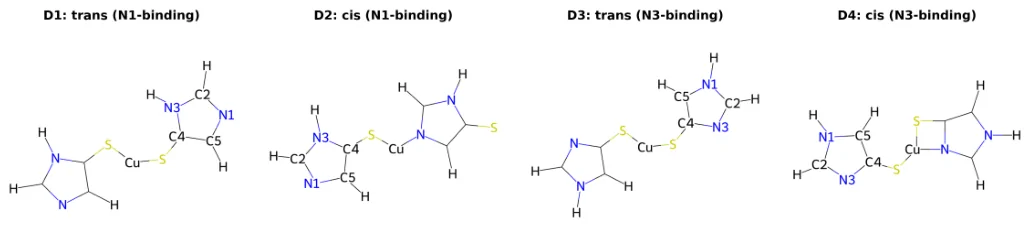
Two-dimensional depictions of the optimized structures of the four Cu(II) isomers: D1 (*trans*, *N1*-binding), D2 (*cis*, *N1*-binding), D3 (*trans*, *N3*-binding), and D4 (*cis*, *N3*-binding). D1/D2 vs. D3/D4 are linkage isomers: the ligand binds copper through *N1* vs. *N3* and accordingly carries its N–H hydrogen on *N3* vs. *N1*; *cis* vs. *trans* denotes the relative orientation of the two ligands. Coordination bonds reflect the optimized geometries (Section 3.5); ring numbering (*N1*, C2, *N3*, C4, C5, S) is labelled on the first ligand of each panel.

Four Cu(II) isomers were optimized, arising from the combination of *cis*/*trans* geometry and *N1*/*N3* nitrogen binding (Table 3; Figure 2). Each isomer is a neutral complex of the formula Cu(II)(4MC^−^)_2_, in which one Cu(II) centre is bound to two monoanionic thione-anion ligands (1:2 Cu:ligand stoichiometry). Both ligands were modelled in a bidentate (S,N) chelation mode, and the optimized structures display graded coordination behaviour: the *trans* isomers (D1, D3) show symmetric, near-linear S–Cu–S arrangements; the dominant *cis*-(*N3*) isomer D4 features one tight S,N chelate and one more loosely associated ligand, while D2 exhibits one effectively dissociated Cu–S contact (5.776 Å) and hence an essentially monodentate coordination (Section 3.5). Figure 2 shows two-dimensional depictions of the optimized structures of the four isomers. No explicit solvent molecules are included in the models; solvation is treated with the IEFPCM continuum only, so the Cu(II) coordination sphere is not complemented by explicit water ligands—a limitation of the present model to keep in mind when comparing with experimental speciation. The *cis*-(*N3*) isomer D4 is the global minimum, with a Boltzmann population of 81.0%. The *trans*-(*N3*) isomer D3 is the only other significantly populated isomer at 19.0%. The *N1*-binding isomers (D1, D2) are more than 29 kJ mol^−1^ higher in energy and have negligible populations (<0.001%).

The strong preference for *N3*-binding in the Cu(II) complexes mirrors the tautomer and anion preferences: since the *N3*-H thione anion is the preferred anion, copper coordination through *N1* (which bears the negative charge) is the natural binding mode. The *cis* geometry of D4 likely provides better orbital overlap for the four-membered chelate ring (Cu–S–C–N) compared to the *trans* arrangement.

**Figure 3:**
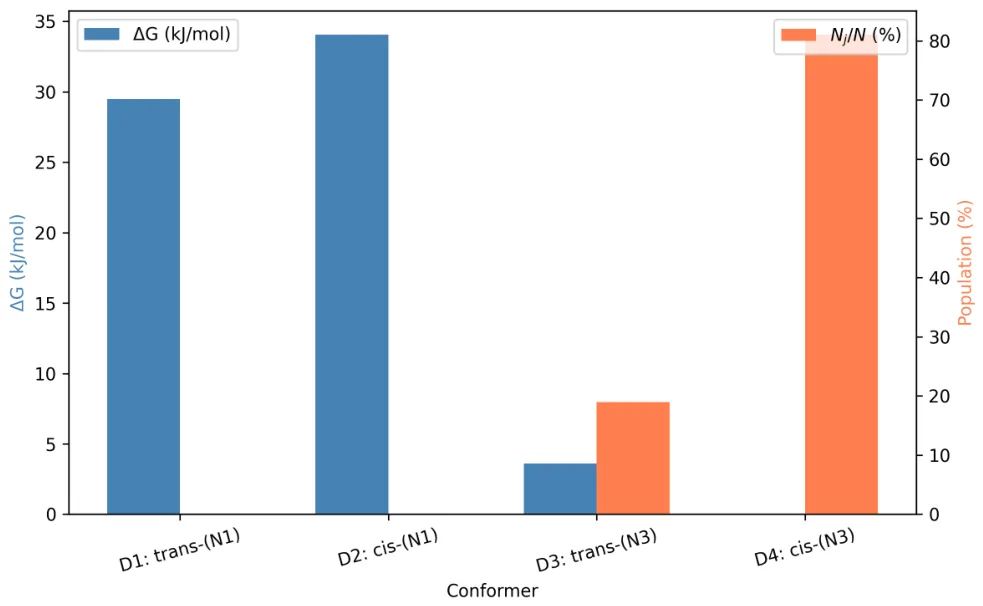
Boltzmann populations of the four Cu(II) isomers at 298.15 K. Only the *N3*-binding isomers (D3, D4) are significantly populated.

Figure 3 visualises the Boltzmann population distribution and highlights the overwhelming dominance of the two *N3*-binding isomers. The *N1*-binding isomers (D1, D2) are essentially absent at room temperature, as their 29 kJ mol^−1^ energy penalty is too large to be overcome by thermal populations. The *cis*/*trans* energy difference among the *N3* isomers is only 3.60 kJ mol^−1^ (D3 vs. D4), which is modest but sufficient to produce an 81:19 population split. This small gap suggests that the *trans*-(*N3*) isomer D3 may still contribute to the redox chemistry, and accordingly, the Cu(II)/Cu(I) reduction potentials are reported both for individual isomers and as a Boltzmann-weighted average (Section 3.4). The preference for *cis* over *trans* can be rationalised by the chelate effect: in the *cis* arrangement, the two bidentate ligands can both coordinate to copper on the same face, stabilising the four-coordinate geometry, whereas the *trans* arrangement imposes greater strain on the Cu–S–C–N chelate rings.

### 3.4. Reduction Potentials

Using the dominant thione tautomer (L2), the thione disulfide (L6), and the dominant Cu(I) complex (C2, thione), the reduction potentials were computed via Equations (6) and (9) (Table 4).

The disulfide/thiol reduction potential $E^{\circ }=0.286\;V$ falls within the literature range for ovothiol-type disulfide/thiol couples (0.25 V to 0.70 V; [10,12]). The Boltzmann-weighted Cu(II)/Cu(I) complex reduction potential $E^{\circ }=0.828\;V$ is lower than all ovothiol analogues (1.15 V to 1.39 V), as discussed in Section 3.7. The resulting EMF is:

(14) $$\mathrm{EMF}=E_{\mathrm{Cu}(\mathrm{II})/\mathrm{Cu}(I)}^{\circ }-E_{\mathrm{disulfide}/\mathrm{thiol}}^{\circ }=0.828-0.286=0.542\;V$$

The positive EMF confirms that the coupled reaction, Cu(II) reduction to Cu(I) with concomitant disulfide formation, is thermodynamically favourable.

**Figure 4:**
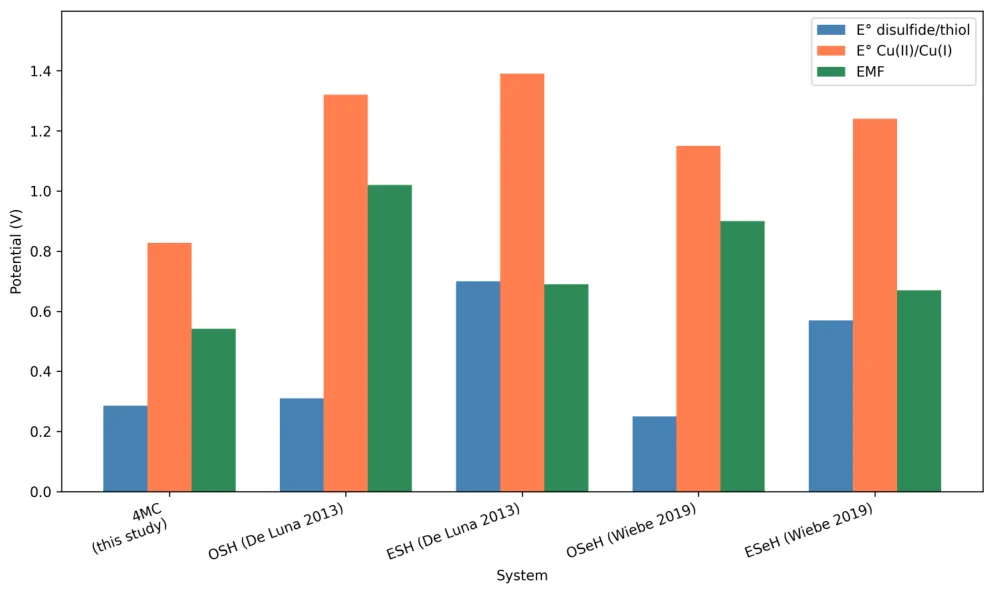
Comparison of calculated reduction potentials for 4MC with literature values for ovothiol (OSH), ergothioneine (ESH), and their selenium analogues (OSeH, ESeH) [10,12]. Top: disulfide/thiol $E^{\circ }$; bottom: Cu(II)/Cu(I) complex $E^{\circ }$.

Figure 4 compares the calculated reduction potentials of 4MC with the literature values for ovothiol (OSH), ergothioneine (ESH), and their selenium analogues (OSeH, ESeH). The disulfide/thiol potential of 4MC (0.286 V) sits in the lower portion of the range, close to OSeH and OSH, while the Cu(II)/Cu(I) complex potential (0.828 V) is visibly lower than all four analogues, which cluster between 1.15 V to 1.39 V. This systematic offset in the copper potential is addressed in detail in Section 3.7, where it is quantified by an M06-2X functional benchmark and decomposed into a functional contribution and a genuine scaffold effect (absence of the trimethylammonium side chain).

**Figure 5:**
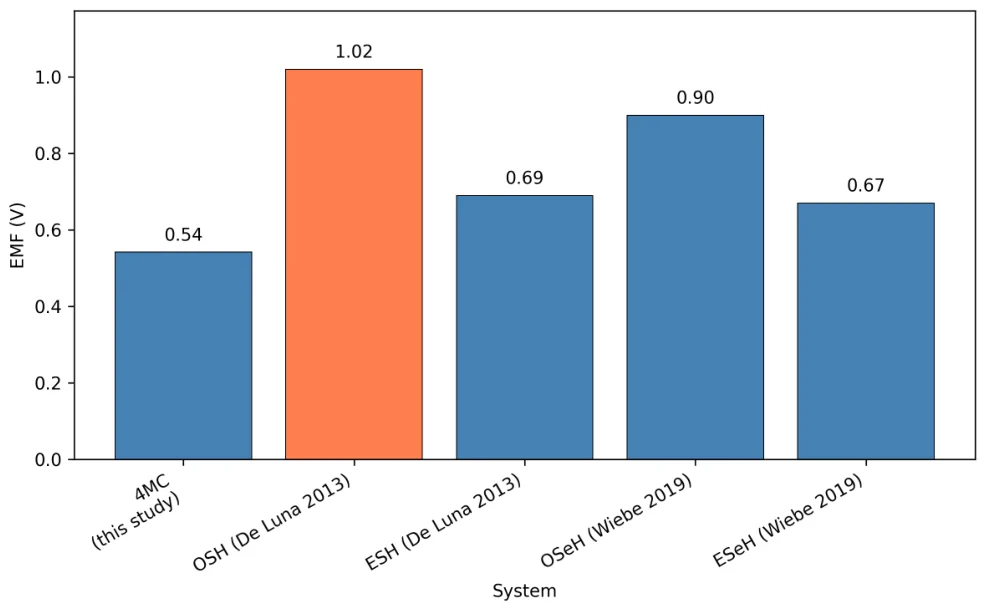
Comparison of EMF values. 4MC (this study) is compared with OSH, ESH, OSeH, and ESeH [10,12]. The positive EMF for all systems confirms thermodynamically favourable redox cycling.

Figure 5 presents the corresponding EMF values. Although the Cu(II)/Cu(I) potential of 4MC is lower than the ovothiol analogues, the disulfide/thiol potential is also lower, and the difference (the EMF) remains positive and substantial (0.542 V). The EMF of 4MC is comparable to ESH (0.690 V) and ESeH (0.670 V), confirming that the thermodynamic driving force for copper-mediated disulfide formation is preserved in the minimal scaffold. A detailed comparison is provided in Section 3.7.

### 3.5. Structural Parameters

Selected structural parameters for the copper complexes are summarised in Table 5; two-dimensional depictions of the four Cu(II) isomers are shown in Figure 2 (Section 3.3), and the Cartesian coordinates of all optimized geometries are provided in Table S13 of the Supplementary Information.

The Cu–S bond distances range from 2.15 Å to 2.39 Å for the well-bound ligands, consistent with Cu–thiolate coordination in the literature [7,10]. The *trans* isomers (D1, D3) display idealised linear S–Cu–S (180^∘^) with symmetric Cu–S distances, while the dominant *cis* isomer D4 shows asymmetric Cu–S distances (2.189 and 2.394 Å) and asymmetric Cu–N distances (2.898 and 2.009 Å), suggesting one well-bound and one more loosely associated ligand. The D2 isomer exhibits one severely elongated Cu–S2 distance (5.776 Å), indicating effective monodentate coordination in the *cis*-(*N1*) geometry.

These geometric features reflect the expected coordination chemistry of the two oxidation states. The two Cu(I) complexes (C1, C2) are tricoordinate and approximately trigonal planar, as expected for d^10^ copper(I) [7]: C2 is bound by three thione sulfur donors, while C1—whose three ligands retain their thiol S–H bonds—is bound by two sulfur and one nitrogen donor, with the third sulfur forming only a weak secondary Cu···S contact (3.073 Å). In contrast, the Cu(II) complexes (D1–D4) display a more distorted geometry characteristic of the Jahn–Teller-active d^9^ configuration. The *trans* isomers (D1, D3) show symmetric, near-linear S–Cu–S arrangements with the two ligands on opposite sides of the copper centre, while the *cis* isomers (D2, D4) fold the two ligands onto the same side, creating a more compact but strained geometry. In D4, the dominant isomer, the asymmetry of the two Cu–S bonds is evident: one ligand is tightly bound (2.189 Å) while the other is more loosely associated (2.394 Å), and the weak Cu–N1 contact (2.898 Å) in D4 reflects the long-range interaction with the deprotonated nitrogen. The D2 structure is the most striking, with one sulfur essentially dissociated (5.776 Å), leaving copper in an effectively two-coordinate environment that helps explain its high energy and negligible Boltzmann population.

### 3.6. Spin Contamination

All four Cu(II) doublets show minimal spin contamination, with $\langle S^{2}\rangle$ values of 0.7532 (D1), 0.7534 (D2), 0.7523 (D3), and 0.7523 (D4), compared to the ideal value of 0.7500 for a pure S=1/2 state. This confirms that the unrestricted Kohn–Sham determinant is a good approximation to the true doublet state for all Cu(II) species.

The largest deviation from the ideal value is only 0.0034 (D2), well below the commonly cited threshold of 0.01 above which spin contamination begins to significantly affect computed energetics and geometries [35]. Such low contamination is typical for Cu(II) (d^9^) complexes, where the unpaired electron occupies a well-localised metal-centred orbital and there is minimal spin polarisation onto the ligands. This is consistent with the electronic structure analysis in Section 3.9, which shows that the spin density is predominantly copper-centred.

The practical consequence is that the unrestricted DFT framework used throughout this study provides reliable single-point energies and, by extension, reliable reduction potentials for the Cu(II)/Cu(I) couple. No spin-projection or spin-purification correction [36] was necessary, as the contamination is too small to introduce meaningful errors into the Boltzmann-weighted potentials reported in Section 3.4.

### 3.7. Comparison with Ovothiol Literature and Minimal Scaffold Analysis

Table 6 compares the key redox parameters of 4MC with those of the naturally occurring ovothiols (OSH, ESH) and their selenium analogues (OSeH, ESeH) from the DFT studies of De Luna et al. [10] and Wiebe et al. [12].

For the disulfide/thiol potential, the $E^{\circ }=0.286\;V$ for 4MC falls in the lower portion of the literature range (0.25 V to 0.70 V), being closest to OSeH (0.250 V) and OSH (0.310 V). This is consistent with 4MC being the minimal parent scaffold: the absence of the amino acid side chain and the quaternary ammonium group reduces the stabilisation of the reduced (thiol) form relative to the oxidised (disulfide) form, lowering the reduction potential. However, the core reactivity is preserved, as 4MC remains well within the biologically relevant range for thiol/disulfide redox [9].

For the Cu(II)/Cu(I) complex potential, the $E^{\circ }=0.828\;V$ for 4MC is lower than all ovothiol analogues (1.15 V to 1.39 V). To separate the contribution of the computational protocol from that of the minimal scaffold itself, the key parent species (L2, L6, C2, and the four Cu(II) isomers) were additionally evaluated at the M06-2X/def2-TZVP/IEFPCM level on the same BP86/def2-SVP geometries (Table 7). The choice of functional has a large and quantifiable effect: it raises the Boltzmann-weighted Cu(II)/Cu(I) potential by 0.236 V (from 0.828 V to 1.063 V), with the Boltzmann population ordering unchanged (D4 dominant at both levels), and lowers the disulfide/thiol potential by 0.126 V (from 0.286 V to 0.160 V), giving an EMF of 0.904 V. A substantial part of the offset between the 4MC copper potential and the ovothiol literature values is therefore attributable to the functional: BP86, a pure GGA with a different self-interaction error profile which systematically underestimates this reduction potential. The remaining gap of 0.09 V to 0.33 V after the M06-2X correction is the genuine scaffold effect: (i) the absence of the quaternary ammonium side chain in 4MC, which in the full ovothiols stabilises Cu(I) through electrostatic interactions with the anionic carboxylate; (ii) the Boltzmann-weighted average includes contributions from both *N1* and *N3* binding isomers, though the *N1* isomers have negligible weight at both levels of theory.

Despite the lower absolute value, the key qualitative finding is preserved: the copper complex potential (0.828 V) is well above the aqueous Cu(II)/Cu(I) potential (0.160 V), indicating that 4MC strongly stabilises Cu(I) relative to Cu(II), as expected for thiolate coordination [7,8].

For the EMF and redox cycling, the EMF of +0.542 V for 4MC confirms that the coupled Cu(II)-to-Cu(I) reduction with disulfide formation is thermodynamically favourable. This EMF is lower than OSH (1.020 V) and OSeH (0.900 V) but comparable to ESH (0.690 V) and ESeH (0.670 V). This places 4MC in the same reactivity regime as the ovothiols, demonstrating that the minimal mercaptoimidazole scaffold retains the essential thermodynamic driving force for copper-mediated thiol/disulfide redox cycling. This ligand–redox coupling is also of contemporary relevance beyond the ovothiol family: recent studies of copper- and photocatalytic synergistic radical cyclization strategies [37] and of Cu-catalyzed radical C–C triple-bond cleavage and amidation [38] highlight how the Cu(I)/Cu(II) redox couple and the choice of ligand jointly govern reactivity in modern copper catalysis, underscoring the value of understanding how a minimal ligand such as 4MC modulates the copper reduction potential.

Regarding 4MC as a minimal parent scaffold, the central finding of this study is that 4MC, the smallest possible member of the ovothiol/ergothioneine family, exhibits the same qualitative redox chemistry as its naturally occurring derivatives. The thione tautomer dominance ($\Delta G_{\mathrm{taut}}=-2.56\;\mathrm{kJ}\;{\mathrm{mol}}^{-1}$), the *N3*-H anion preference ($\Delta G_{\mathrm{anion}}=-14.63\;\mathrm{kJ}\;{\mathrm{mol}}^{-1}$), the *N3*-binding selectivity for Cu(II) (81% *cis*-*N3* population), and the favourable EMF all mirror the behaviour of the full ovothiols.

The lower Cu(II)/Cu(I) reduction potential (0.828 V vs. 1.15 V to 1.39 V) suggests that the additional structural features of the ovothiols, particularly the trimethylammonium group and the amino acid side chain, serve to fine-tune the copper-binding affinity upward, rather than fundamentally alter the redox mechanism. This is consistent with the design principle that the mercaptoimidazole core is the pharmacophore, while the peripheral groups provide optimisation [20]. From a practical standpoint, this validates 4MC as a useful model compound for studying ovothiol-type redox chemistry and as a potential scaffold for the design of simplified antioxidant agents [21,22].

### 3.8. Substituent Effects: Hammett Analysis

To assess how electronic perturbations at the C2 position modulate the redox chemistry of the 4-mercaptoimidazole (4MC) scaffold, three C2-substituted derivatives were studied: CH_3_ ($\sigma_{p}=-0.17$, electron-donating), Cl ($\sigma_{p}=+0.23$, weakly electron-withdrawing), and NO_2_ ($\sigma_{p}=+0.78$, strongly electron-withdrawing) (Table 8). For each substituent, five species were computed: the thione tautomer, the N3-H thione anion, the homodimeric disulfide, the Cu(I) complex, and the dominant D4 *cis*-*N3* Cu(II) isomer, mirroring the parent 4MC protocol (Section 3.1, Section 3.2, Section 3.3 and Section 3.4).

Note that the parent H row uses the D4-only $E_{\mathrm{Cu}}^{\circ }=0.821$ V (rather than the Boltzmann-weighted 0.828 V reported in Section 3.4) for consistency with the derivative protocol, which computes only the D4 isomer; this gives a parent EMF of 0.535 V, slightly lower than the Boltzmann-weighted value of 0.542 V.

The Hammett equation, $E^{\circ }=\rho \;\sigma_{p}+E_{0}^{\circ }$, was fitted to the four data points (H, CH_3_, Cl, NO_2_) for each redox couple (Figure S1 in the Supplementary Materials). The regression parameters are summarised in Table 9.

The disulfide/thiol couple is essentially insensitive to C2 substitution (ρ=−0.044 V, $R^{2}=0.039$): the three substituted derivatives span only 0.109–0.315 V, and CH_3_ and NO_2_ deviate from the parent value by less than 0.03 V. This weak response is consistent with the redox-active C4–S–S–C4 unit being separated from the C2 substituent position so that through-bond electronic communication is strongly attenuated. The Cu(II)/Cu(I) couple shows a nominally larger sensitivity (ρ=+0.134 V) but essentially no linear correlation ($R^{2}=0.045$), suggesting that substituent effects on the copper redox are dominated by factors beyond through-bond electronics, likely geometric reorganisation of the N_3_–Cu coordination sphere and through-space interactions that vary nonlinearly with substituent size and polarity.

All four substituents retain a positive EMF for copper-catalysed disulfide formation (0.302–0.738 V), indicating that the thermodynamic coupling between the thiol–disulfide and copper centres persists across C2 substitution. The EMF varies non-monotonically with $\sigma_{p}$ (ρ=+0.178 V, $R^{2}=0.167$): CH_3_ and NO_2_ slightly increase the driving force (+0.586 and +0.738 V vs. +0.535 V for the parent), whereas Cl reduces it (+0.302 V). The chloro derivative is again the outlier: both of its reduction potentials shift downward together ($E_{\mathrm{Cu}}^{\circ }$ drops by 0.41 V and $E_{\mathrm{dis}}^{\circ }$ by 0.18 V relative to the parent), and because the copper couple responds more strongly, the EMF decreases. This co-directional shift shows that C2 substituents tune the two redox centres with different magnitudes rather than independently, and the simple Hammett framework provides only a qualitative guide to the overall trend. The results demonstrate that C2 functionalisation of the 4MC scaffold modulates both the thiol–disulfide and copper redox potentials over a range of ca. 0.4 V in EMF without abolishing the thermodynamic driving force for redox cycling.

The electronic-structure mechanism underlying the pronounced downward shift of the chloro-substituted potentials was examined by extending the NBO analysis to the Cl-substituted copper complexes Cl-C2 and Cl-D4 and to the Cl-substituted disulfide itself (Section 3.9). In the Cu(II) complex, Cl substitution makes the Cu–S bonding markedly more symmetric and more covalent: the two Cu–S Wiberg bond indices become nearly equal (0.139 and 0.134 vs. 0.101 and 0.185 in the parent D4, whose Cu–S distances of 2.189/2.394 Å are strongly asymmetric), and the Cu NPA charge drops from +0.532 to +0.340, indicating substantially enhanced electron donation from the thiolate sulfurs to Cu(II). In contrast, the Cu(I) complex is almost unaffected (Cu NPA charge +0.297 vs. +0.294 in the parent C2; Cu–S Wiberg indices of 0.066–0.078 unchanged). Cl substitution thus stabilises the oxidised Cu(II) state through stronger, more symmetric Cu–S covalency while leaving the reduced Cu(I) state essentially unchanged, which lowers the Cu(II)/Cu(I) reduction potential by 0.41 V. On the disulfide side, the Cl-substituted disulfide is stabilised by ca. 34 kJ mol^−1^ relative to its thione pair compared with the parent, lowering $E_{\mathrm{dis}}^{\circ }$ by 0.18 V; notably, the S–S bond itself is barely perturbed (Wiberg bond index 0.217 vs. 0.221 in the parent L6; sulfur NPA charges +0.067/+0.069 vs. +0.064/+0.065), indicating that this stabilisation is a ring-level substituent effect transmitted to the C4–S linkage rather than a weakening of the S–S bond. Both redox couples, therefore, shift downward for Cl, the copper couple more strongly, which accounts for the reduced but still positive EMF (+0.302 V).

For the thermodynamic driving forces, Table 10 collects the reaction free energies ($\Delta G^{\circ }$) underlying the reduction potentials in Table 8. Both the disulfide/thiol and Cu(II)/Cu(I) half-reactions are thermodynamically favourable (negative $\Delta G^{\circ }$) for all four substituents. The disulfide reduction free energy is nearly substituent-independent (−21.0 to −60.7 kJ mol^−1^, vs. −55.2 kJ mol^−1^ for the parent H), with CH_3_ slightly more exergonic and Cl the least exergonic; the absence of a systematic electron-withdrawing trend mirrors the near-zero Hammett slope of this couple. The copper reduction shows a different trend: the parent H has $\Delta G^{\circ }=-158.3$ kJ mol^−1^, while Cl has the smallest magnitude (−79.3 kJ mol^−1^), explaining its anomalously low $E_{\mathrm{Cu}}^{\circ }$.

The overall EMF remains positive for all four substituents (0.302–0.738 V), meaning that copper-catalysed disulfide formation is thermodynamically favourable across the whole series. C2 substitution, therefore, tunes the magnitude of the driving force—spanning a range of ca. 0.44 V between the Cl (+0.302 V) and NO_2_ (+0.738 V) derivatives—without disrupting the thermodynamic coupling between the thiol–disulfide and copper centres that is essential for catalytic disulfide formation. Combined with the weak Hammett correlations of the individual couples, this indicates that the substituent effects are dominated by system-specific electronic–structural responses (such as the symmetric Cu–S covalency induced by Cl) rather than by a uniform through-bond electronic effect.

For the structural parameters of the derivative copper complexes, Table 11 reports the Cu–S bond distances for the derivative Cu(I) and Cu(II) complexes alongside the parent values. In the trigonal-planar Cu(I) complexes, the mean Cu–S distance is remarkably conserved across all four substituents (2.28–2.29 Å), indicating that the C2 substituent has minimal influence on the Cu(I) coordination geometry. The individual Cu–S bonds within each complex vary by up to 0.08 Å, reflecting the asymmetric ligand arrangement, but the average is essentially substituent-independent.

In contrast, the Cu(II) D4 complexes show a striking structural response to substitution. The parent D4 has a markedly asymmetric Cu–S coordination (2.189 vs. 2.394 Å), with the shorter bond corresponding to the *N3*-coordinated ligand and the longer bond to the non-*N3* ligand. The CH_3_ and Cl derivatives both show much more symmetric Cu–S distances (2.164/2.166 and 2.164/2.169 Å, respectively), suggesting that the substituent relieves the asymmetry present in the parent. The shorter Cu–S distances in the Cu(II) complexes compared to Cu(I) are consistent with the higher oxidation state and the stronger Cu–S bonding reflected in the Wiberg bond indices (Section 3.9).

The NO_2_-substituted Cu(II) complex exhibits a qualitatively different coordination mode: one Cu–S bond is maintained at 2.162 Å, but the second Cu–S distance elongates to 6.318 Å, indicating complete bond dissociation. Instead, a Cu–N contact at 2.766 Åis formed, suggesting that the strongly electron-withdrawing NO_2_ group destabilises the Cu–S coordination to the point where the ligand reorients to coordinate through nitrogen. This structural disruption is consistent with the extremely low Wiberg bond index (0.017) for this Cu–S interaction and the absence of a corresponding QTAIM bond critical point (Section 3.9). The NO_2_-induced coordination change represents an extreme case of substituent effects on the copper coordination sphere and highlights the limit of the Hammett model, which assumes a uniform structural response across the series.

### 3.9. Electronic Structure Analysis

**Figure 6:**
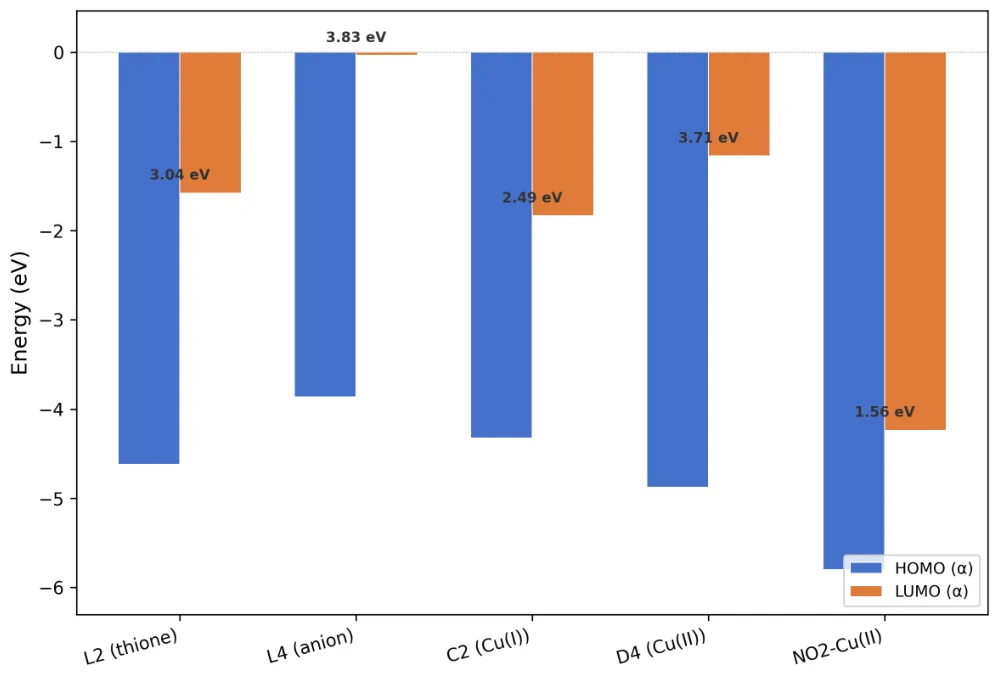
HOMO and LUMO energies for the five key species. HOMO–LUMO gaps (eV) are annotated above each pair. The Cu(I) complex C2 has the smallest gap among closed-shell species, while the open-shell Cu(II) species exhibit very small β-orbital gaps.

**Figure 7:**
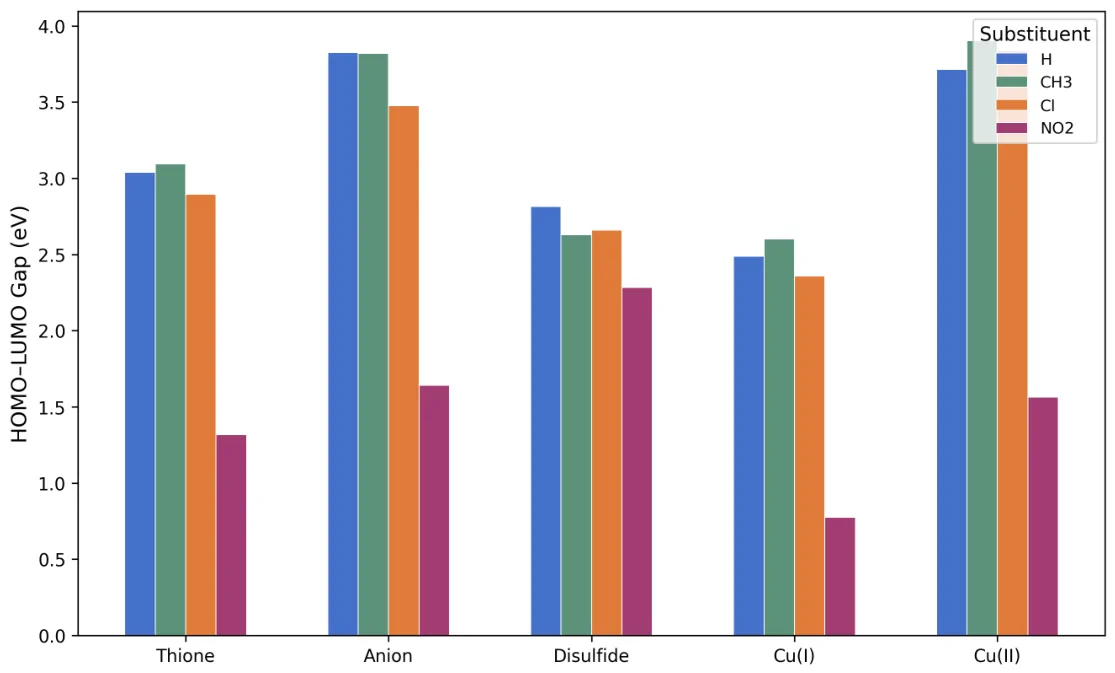
HOMO–LUMO gaps grouped by species category and C2 substituent. The NO_2_ substituent (purple) dramatically reduces the gap across all categories, particularly for the Cu(I) and Cu(II) complexes.

**Figure 8:**
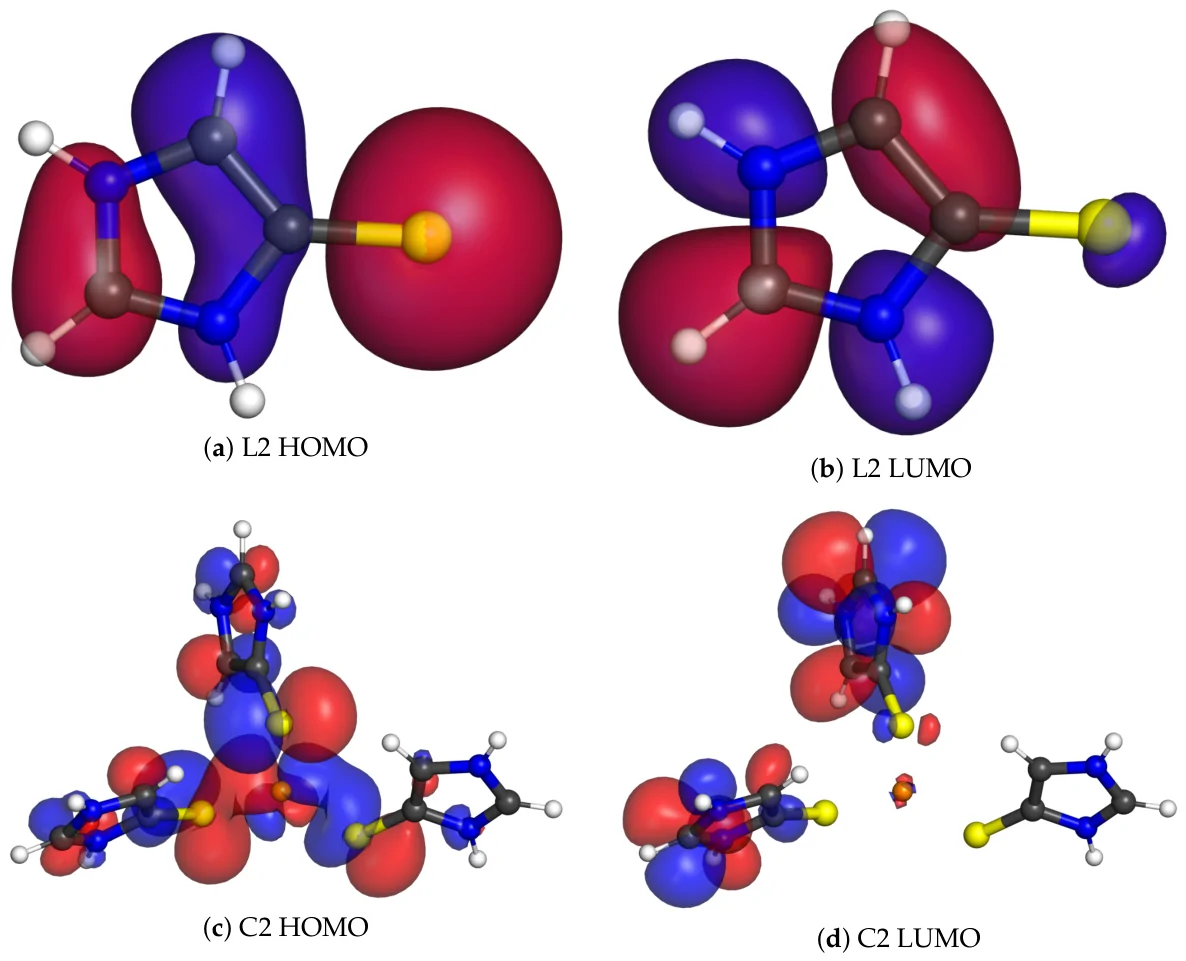
3D isosurface plots (|*iso*| = 0.02 a.u.) of the frontier orbitals for the dominant thione tautomer L2 (**top row**) and the Cu(I) complex C2 (**bottom row**). Red and blue surfaces denote positive and negative orbital phases, respectively. The molecular structure is shown as CPK-coloured spheres (C gray, N blue, S yellow, Cu orange, H white). In C2, the HOMO has significant Cu–S character and the LUMO is localised on the metal centre.

**Figure 9:**
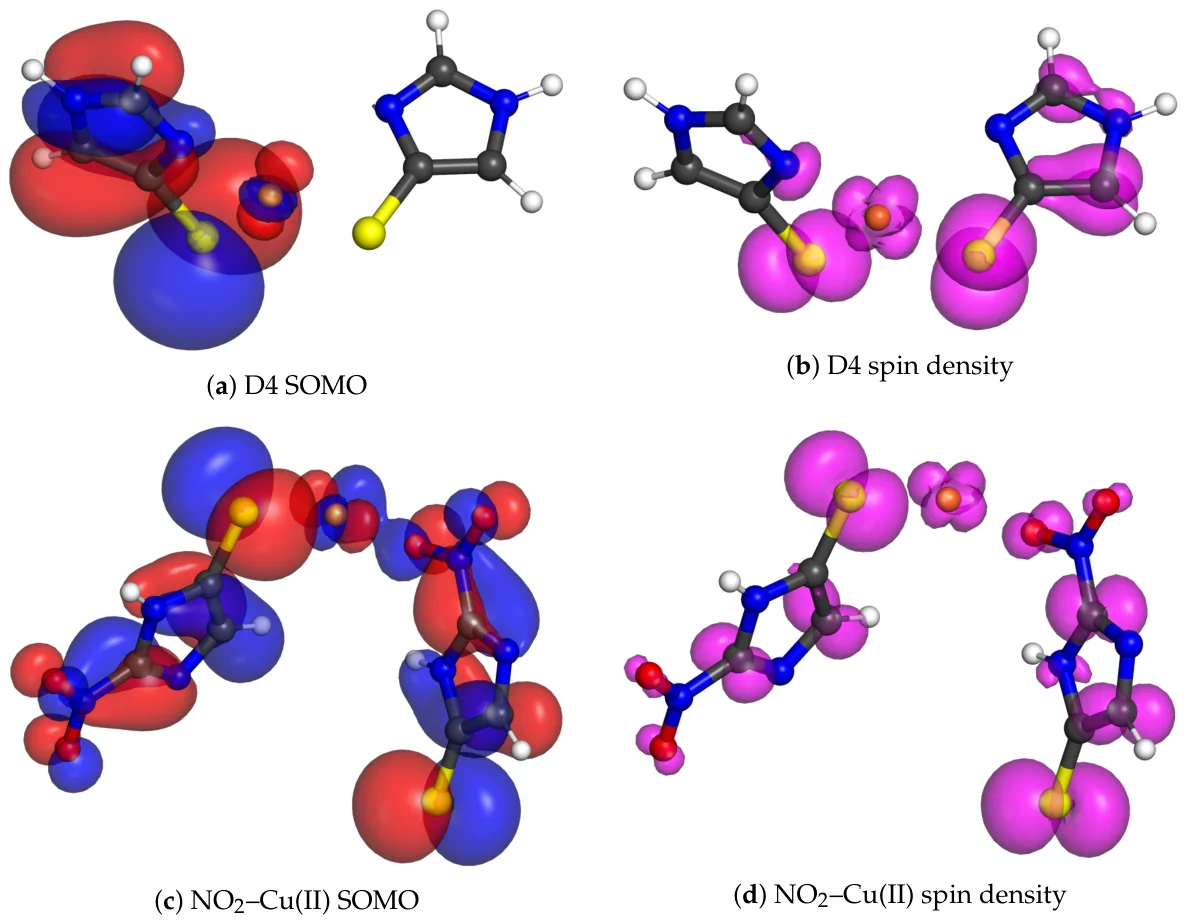
3D isosurface plots of the singly occupied molecular orbital (SOMO, |*iso*| = 0.02 a.u., left column of each pair) and spin density (|*iso*| = 0.002 a.u., right column) for the Cu(II) species D4 (**top**) and NO_2_–Cu(II) (**bottom**). The SOMO (red/blue) is delocalised over the Cu–S framework, and the spin density (magenta) confirms substantial ligand contribution, consistent with the Mulliken spin populations in Table 16.

**Figure 10:**
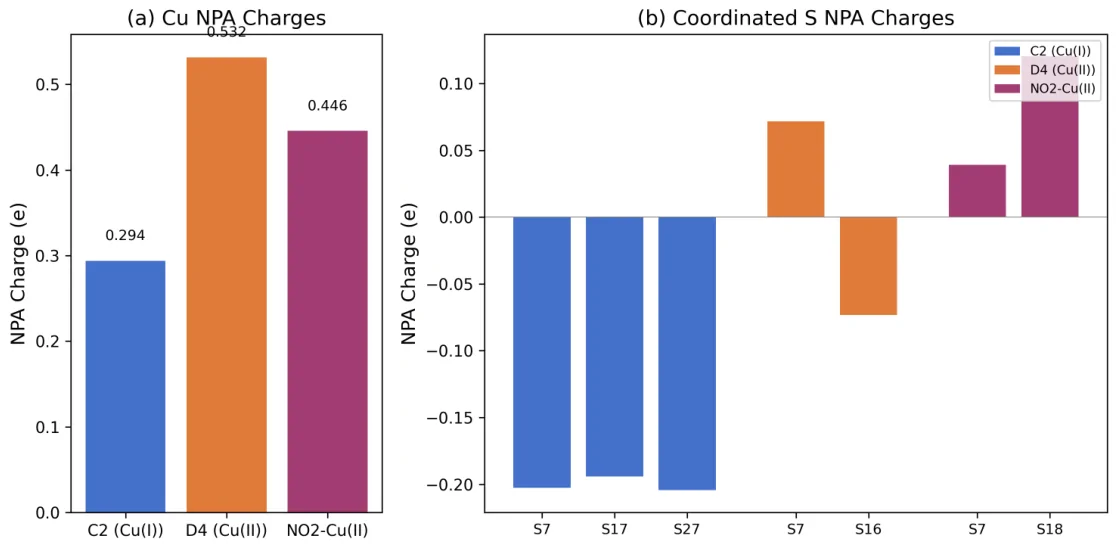
NPA charges on (**a**) the Cu atom and (**b**) coordinated S atoms for the three copper complexes. Cu NPA charge increases with oxidation state; S charges become less negative upon Cu(II) coordination, reflecting significant electron donation.

**Figure 11:**
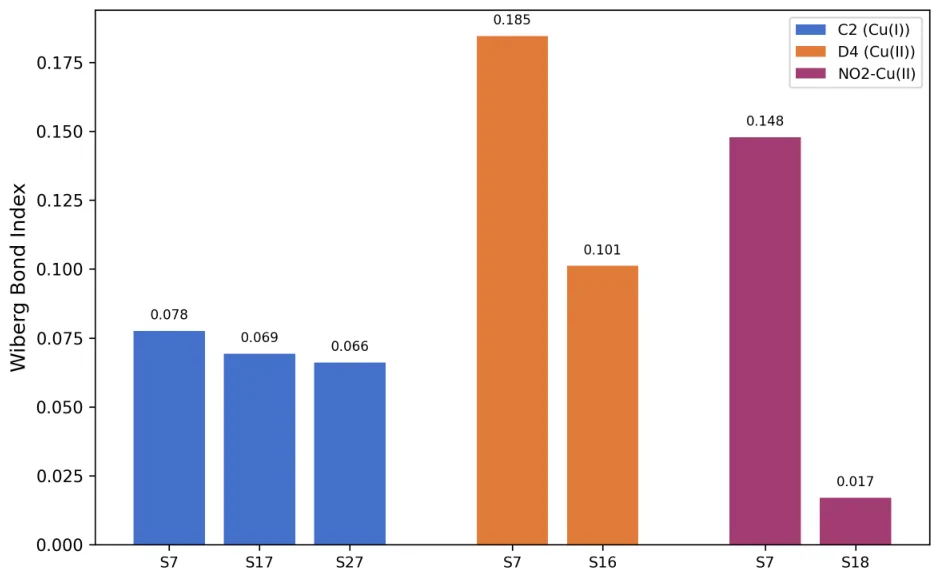
Cu–S Wiberg bond indices. The Cu(II) species D4 (orange) shows stronger Cu–S bonds than the Cu(I) species C2 (blue). The NO_2_-substituted Cu(II) complex (purple) shows highly asymmetric bonding.

**Figure 12:**
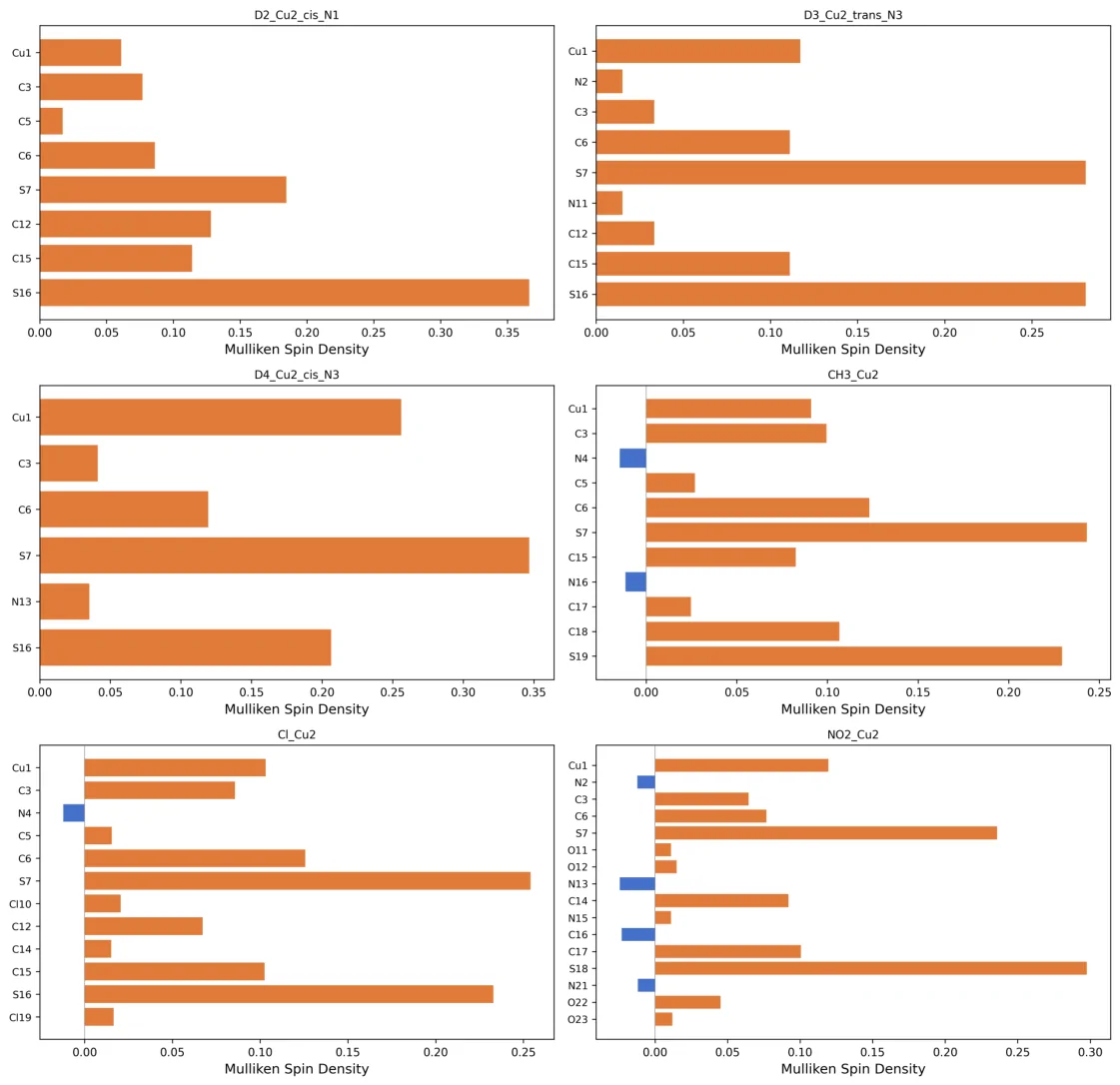
Mulliken spin density distribution for the open-shell Cu(II) species D4 (**left**) and NO_2_–Cu(II) (**right**). Positive spin density (orange) is delocalised over Cu and S atoms, indicating significant covalent character in the Cu–S bonding.

**Figure 13:**
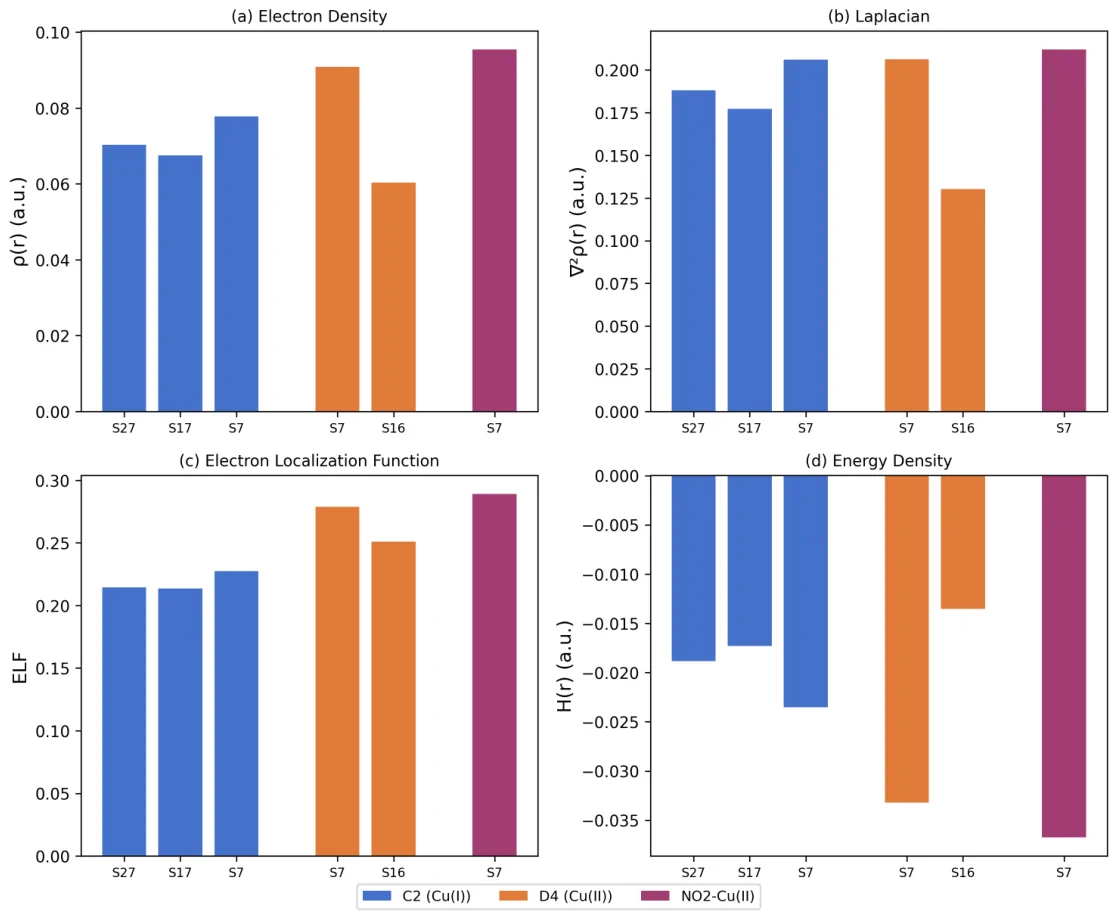
QTAIM bond critical point properties for Cu–S bonds: (**a**) electron density ρ(r), (**b**) Laplacian $\nabla^{2}\rho \left(r\right)$, (**c**) electron localisation function (ELF), and (**d**) energy density H(r). Blue: C2 (Cu(I)); orange: D4 (Cu(II)); purple: NO_2_–Cu(II). All BCPs show positive $\nabla^{2}\rho$ (closed-shell) but negative H(r) (partial covalency).

To characterise the bonding and electronic structure of the key species in the 4MC–Cu redox cycle, additional single-point calculations were performed at the BP86/def2-TZVP/IEFPCM(water) level for five representative species: the dominant thione tautomer L2, the preferred anion L4, the dominant Cu(I) complex C2, the dominant Cu(II) isomer D4, and the NO_2_-substituted Cu(II) complex. To characterize the electronic-structure origin of the Cl outlier in the Hammett series (Section 3.8), the NBO analysis was extended to the Cl-substituted copper complexes Cl-C2 and Cl-D4 and to the parent and Cl-substituted disulfides (L6 and Cl-L6). The analysis includes frontier orbital energies, Natural Population Analysis (NPA) charges, Wiberg bond indices, spin densities, and Quantum Theory of Atoms in Molecules (QTAIM) topological descriptors. The electronic structure data are summarised in Table 12, Table 13, Table 14, Table 15, Table 16 and Table 17 and Figure 6, Figure 7, Figure 8, Figure 9, Figure 10, Figure 11, Figure 12 and Figure 13.

For the frontier orbital energies, the HOMO–LUMO gaps (Figure 6, Table 12) reveal a clear trend: the Cu(I) complex C2 has the smallest gap (2.49 eV) among the closed-shell species, consistent with its high reactivity as an electron donor in the redox cycle. The free thione L2 (3.04 eV) and anion L4 (3.83 eV) have larger gaps, reflecting their relative stability. The open-shell Cu(II) species D4 and NO_2_–Cu(II) exhibit very small β-HOMO–β-LUMO gaps (0.31 and 0.18 eV, respectively), arising from the low-lying unoccupied β spin–orbital adjacent to the singly occupied molecular orbital (SOMO). The NO_2_ substituent dramatically reduces the HOMO–LUMO gap across all species categories (Figure 7), with the NO_2_-Cu(I) complex showing a particularly small gap of 0.77 eV, indicating that the electron-withdrawing nitro group significantly destabilises the frontier orbitals.

To provide a spatial picture of the frontier orbitals, we visualised the HOMO, LUMO, and singly occupied molecular orbital (SOMO) isosurfaces for the key species using PyMOL (Version 2.5.) [40] at an isovalue of |0.02| a.u. (orbitals) and |0.002| a.u. (spin density). The orbital and spin density data were extracted from the BP86/def2-TZVP wavefunctions using Multiwfn [30]. The molecular structure is rendered as CPK-coloured spheres (C gray, N blue, S yellow, Cu orange, H white) and is visible through the semi-transparent isosurfaces.

Figure 8 shows the frontier orbitals of the free thione L2 and the Cu(I) complex C2. In L2, the HOMO is localised on the sulfur atom and the π-system of the ring, while the LUMO is delocalised over the ring framework. Upon coordination to Cu(I) in C2, the HOMO acquires significant Cu–S antibonding character, and the LUMO is localised on the metal centre, consistent with the small HOMO–LUMO gap (2.49 eV) and the role of Cu(I) as an electron donor.

Figure 9 shows the SOMO and spin density for the Cu(II) species D4 and NO_2_–Cu(II). The SOMO of D4 is delocalised over the Cu–S framework, consistent with the Mulliken spin density analysis (Table 16). The spin density isosurface (Figure 9b) clearly shows the delocalisation over Cu and the coordinated sulfur atoms, rather than being confined to the Cu *d*-orbitals. In NO_2_–Cu(II), the SOMO is further delocalised onto the NO_2_-substituted ligand, consistent with the more dispersed spin population (Table 16).

For the substituent effects on frontier orbitals, the HOMO–LUMO gaps for all 15 derivative species (Table 13) reveal a systematic trend: the strongly electron-withdrawing NO_2_ group dramatically reduces the gap across all five species categories, while CH_3_ and Cl produce more modest changes relative to the parent. For the closed-shell thione, anion, and Cu(I) species, the NO_2_ substituent reduces the gap by 1.4–1.7 eV compared to the parent, reflecting the introduction of low-lying $\pi^{*}$ orbitals localised on the nitro group. The CH_3_ substituent is nearly innocuous (gap changes ≤0.18 eV), while Cl produces small but noticeable gap reductions (0.13–0.35 eV), consistent with their weaker electronic effects.

For the disulfide species, the parent L6 has a gap of 2.81 eV, and the C2-substituted disulfides show only modest changes: 2.63 eV (CH_3_), 2.66 eV (Cl), and 2.28 eV (NO_2_). The largest reduction, for NO_2_, again reflects the low-lying nitro $\pi^{*}$ acceptor orbital lowering the LUMO (from −2.99 eV in the parent to −4.22 eV). The insensitivity of the disulfide gap to CH_3_ and Cl substitution mirrors the near-zero Hammett slope of the disulfide/thiol couple (Section 3.8): the frontier orbitals of the C4–S–S–C4 unit are largely decoupled from the C2 position.

For the open-shell Cu(II) complexes, the α HOMO–LUMO gaps follow the same trend (NO_2_: 1.56 eV vs. parent: 3.71 eV), while the β gaps remain small for all substituents (0.18–0.37 eV), characteristic of the low-lying unoccupied β spin–orbital adjacent to the singly occupied molecular orbital (SOMO).

The Natural Population Analysis (NPA) charges from the NBO analysis (Table 14, Figure 10) reveal the charge redistribution upon copper coordination and oxidation. The Cu NPA charge increases from +0.294 in the Cu(I) complex C2 to +0.532 in the Cu(II) complex D4, consistent with the formal oxidation state change. The coordinated sulfur atoms show a dramatic change: in the free thione L2, the sulfur NPA charge is −0.437, but upon coordination to Cu(I) in C2 it becomes approximately −0.20, and in the Cu(II) species D4 and NO_2_–Cu(II), the sulfur charges become near-zero or even positive (+0.07 for S7 in D4 and +0.12 for S18 in NO_2_–Cu(II)), indicating substantial electron donation from sulfur to copper. The NO_2_ substituent further withdraws electron density from the sulfur donors, with both S atoms in NO_2_–Cu(II) carrying positive NPA charges.

The Wiberg bond indices (WBI) for the Cu–S bonds (Table 15, Figure 11) provide a complementary measure of bond strength. The Cu(II) complex D4 has significantly higher Cu–S WBI values (0.101–0.185) than the Cu(I) complex C2 (0.066–0.078), consistent with the shorter Cu–S distances in D4 (2.189–2.394 Å) compared to C2 (2.249–2.326 Å) and reflecting the stronger Cu–S interaction in the oxidised form. The NO_2_–Cu(II) complex shows highly asymmetric Cu–S bonding: the S7–Cu bond (WBI = 0.148) is comparable to the stronger bond in D4, while the S18–Cu bond (WBI = 0.017) is extremely weak, indicating that the NO_2_ substituent disrupts the coordination symmetry. The Cl-substituted Cu(II) complex (Cl-D4) shows the opposite behaviour: markedly more symmetric and stronger Cu–S bonding than the parent D4 (WBI 0.134 and 0.139 vs. 0.101 and 0.185 for the asymmetric 2.189/2.394 Å pair in D4), together with a substantially lower Cu NPA charge (+0.340 vs. +0.532), indicating enhanced electron donation from the thiolate sulfurs to Cu(II). The Cu(I) complex is almost unaffected by Cl substitution (Cu NPA charge +0.297 vs. +0.294; Cu–S Wiberg indices unchanged, Table 14). For the disulfides, the S–S Wiberg index is essentially unaffected by Cl substitution (0.217 in Cl-L6 vs. 0.221 in the parent L6, Table 15), and the sulfur NPA charges are nearly unchanged (+0.067/+0.069 vs. +0.064/+0.065), confirming that the relative stabilisation of the Cl-substituted disulfide inferred from $E_{\mathrm{dis}}^{\circ }$ (Section 3.8) is a ring-level electronic effect rather than a perturbation of the S–S bond itself. The implications of this differential stabilisation of the two copper oxidation states for the Cl outlier in the Hammett series are discussed in Section 3.8.

For the open-shell Cu(II) species, the Mulliken spin density distribution (Figure 12, Table 16) reveals that the unpaired electron is not localised on the copper *d*-orbitals but is substantially delocalised over the sulfur ligands. In D4, the largest spin populations reside on S7 (0.347), Cu (0.256), and S16 (0.207), with additional delocalisation onto the carbon backbone (C6: 0.120). In NO_2_–Cu(II), the spin is even more delocalised: S18 carries the highest spin density (0.298), followed by S7 (0.236) and Cu (0.120), with significant contributions from the NO_2_-substituted ring (C17: 0.101, C14: 0.092). This delocalised spin distribution indicates substantial ligand-to-metal charge transfer and covalent character in the Cu–S bonding, which is not captured by a purely ionic Cu(II) ($d^{9}$) description.

The Quantum Theory of Atoms in Molecules (QTAIM) analysis of the Cu–S bond critical points (BCPs) provides a topological characterisation of the bonding (Table 17, Figure 13). All Cu–S BCPs exhibit positive Laplacians ($\nabla^{2}\rho >0$), classifying them as closed-shell interactions, consistent with dative Cu–S bonding. However, the energy density H(r) is negative for all Cu–S BCPs, indicating some covalent character, a signature of polar covalent bonding intermediate between pure ionic and covalent limits. The electron density at the BCP ($\rho_{\mathrm{BCP}}$) is higher for the Cu(II) species (D4: 0.060–0.091; NO_2_–Cu(II): 0.096) than for the Cu(I) complex C2 (0.068–0.078), confirming stronger Cu–S interactions upon oxidation. Notably, the extremely weak S18–Cu bond in NO_2_–Cu(II) (WBI = 0.017) does not produce a detectable BCP, confirming that this interaction is below the topological bonding threshold.

The electronic structure analysis reveals several key features of the 4MC–Cu redox system: (i) the Cu(I) complex has the smallest HOMO–LUMO gap among closed-shell species, consistent with its role as the active electron donor; (ii) Cu–S bonding is polar covalent, with positive Laplacian but negative energy density at the BCPs; (iii) oxidation to Cu(II) strengthens the Cu–S bonds (higher WBI and $\rho_{\mathrm{BCP}}$) but the unpaired electron is substantially delocalised over the sulfur ligands, not localised on Cu; (iv) the NO_2_ substituent disrupts the coordination symmetry, dramatically reduces the HOMO–LUMO gap, and further delocalises the spin density onto the substituted ligand; and (v) C2 substitution only modestly affects the disulfide HOMO–LUMO gap (2.28–2.66 eV vs. 2.81 eV for the parent), with the largest reduction for NO_2_ arising from its low-lying $\pi^{*}$ acceptor orbital, and leaves the S–S bond order essentially unperturbed. These findings provide a detailed electronic basis for understanding the redox cycling mechanism and substituent effects discussed in the preceding sections.

## 4. Conclusions

This DFT study of 4-mercaptoimidazole–copper redox chemistry, encompassing 27 species (12 parent + 15 C2-substituted derivatives), demonstrates that the unsubstituted 4MC scaffold already encodes the essential ovothiol redox machinery without the amino acid side chain or quaternary ammonium group. The thione tautomer (*N3*-H) dominates in aqueous solution ($\Delta G=-2.56\;\mathrm{kJ}\;{\mathrm{mol}}^{-1}$), and the corresponding *N3*-H anion is preferred by $-14.63\;\mathrm{kJ}\;{\mathrm{mol}}^{-1}$, leaving *N1* free for copper coordination. Among the four Cu(II) isomers, the *cis*-(*N3*) geometry (D4) is the global minimum (81% Boltzmann population), and the resulting Boltzmann-weighted Cu(II)/Cu(I) potential ($E^{\circ }=0.828\;V$) coupled with the disulfide/thiol potential ($E^{\circ }=0.286\;V$) gives a positive EMF of +0.542 V, placing 4MC in the same reactivity regime as the natural ovothiols (EMF 0.67 V to 1.02 V). An M06-2X benchmark on the same geometries shows that roughly half of the residual offset to the ovothiol potentials is functional in origin (the copper potential rises to 1.063 V and the EMF to 0.904 V), with the remainder reflecting the absent trimethylammonium side chain.

Among the C2-substituted derivatives (CH_3_, Cl, NO_2_; $\sigma_{p}$ from −0.17 to +0.78), the disulfide/thiol potential is nearly insensitive to $\sigma_{p}$ (ρ=−0.044 V), whereas the Cu(II)/Cu(I) potential varies over 0.411–1.009 V without a clear Hammett correlation; nevertheless, the EMF remains positive for all four substituents (+0.302 V to +0.738 V), so C2 substitution tunes the driving force of the redox cycle without disrupting the coupling between the two redox centres. These substituent effects arise from system-specific responses, such as the symmetric and more covalent Cu–S bonding that stabilises the Cu(II) state in the Cl derivative, rather than from a uniform through-bond electronic effect.

Electronic structure analysis (NBO, QTAIM) confirms the polar covalent nature of Cu–S bonding (positive $\nabla^{2}\rho$, negative H(r) at the bond critical points; WBI =0.07–0.19 for bonded interactions), with substantial spin delocalisation onto the sulfur ligands in the Cu(II) complexes. C2 substitution leaves the disulfide frontier orbitals and the S–S bond essentially unperturbed (HOMO–LUMO gaps of 2.28–2.66 eV vs. 2.81 eV for the parent), whereas the NO_2_ derivative dissociates one Cu–S bond and switches to mixed Cu–S/Cu–N coordination.

Overall, 4MC retains the essential ovothiol-type redox chemistry within a minimal scaffold, validating the mercaptoimidazole core as the pharmacophore. C2 substitution provides a genuine handle for modulating the driving force of the copper-catalysed redox cycle, but the weak Hammett correlations and the NO_2_-induced coordination switch caution against extrapolating simple substituent trends to derivatised mercaptoimidazoles for antioxidant or metal-binding applications.

## Figures and Tables

**Table 1:** Species studied in this work. The parent set (L1–D4) covers the full tautomeric, protonation, redox, and isomeric landscape of 4MC–copper chemistry. The derivative set adds three C2 substituents (*R* = CH_3_, Cl, NO_2_) with five species each, mirroring the dominant parent forms: thione (L2), anion (L4), disulfide (L6), Cu(I) (C2), and Cu(II) D4.

ID	Description	Atoms	Charge
*Parent species*			
L1	4MC thiol (*N1*-H, C4–SH)	10	0
L2	4MC thione (*N3*-H, C4=S)	10	0
L3	4MC^−^ thiolate (*N1*-H, S^−^)	9	−1
L4	4MC^−^ thione anion (*N3*-H, *N1*^−^)	9	−1
L5	(4MC)_2_ disulfide (thiol, *N1*-H)	18	0
L6	(4MC-*N3*H)_2_ disulfide (thione, *N3*-H)	18	0
C1	Cu(I)(4MC)_3_^+^ (S,N-monodentate, thiol)	31	+1
C2	Cu(I)(4MC-N_3_H)_3_^+^ (S-monodentate, thione)	31	+1
D1	*trans*-Cu(II)(4MC^−^)_2_ (*N1*-binding)	19	0
D2	*cis*-Cu(II)(4MC^−^)_2_ (*N1*-binding)	19	0
D3	*trans*-Cu(II)(4MC^−^)_2_ (*N3*-binding)	19	0
D4	*cis*-Cu(II)(4MC^−^)_2_ (*N3*-binding)	19	0
*C2-substituted derivatives* (*R* at C2)			
*R = CH_3_ ($\sigma_{p}=-0.17$)*			
CH3-L2	2-CH_3_-4MC thione (*N3*-H, C4=S)	13	0
CH3-L4	2-CH_3_-4MC^−^ anion (*N3*-H, *N1*^−^)	12	−1
CH3-L6	(2-CH_3_-4MC-*N3*H)_2_ disulfide	24	0
CH3-C2	Cu(I)(2-CH_3_−4MC−N_3_H)_3_^+^ (S-monodentate)	40	+1
CH3-D4	*cis*-Cu(II)(2-CH_3_−4MC^−^)_2_ (*N3*-binding)	25	0
*R = Cl ($\sigma_{p}=+0.23$)*			
Cl-L2	2-Cl-4MC thione (*N3*-H, C4=S)	10	0
Cl-L4	2-Cl-4MC^−^ anion (*N3*-H, *N1*^−^)	9	−1
Cl-L6	(2-Cl-4MC-*N3*H)_2_ disulfide	18	0
Cl-C2	Cu(I)(2-Cl−4MC−N_3_H)_3_^+^ (S-monodentate)	31	+1
Cl-D4	*cis*-Cu(II)(2-Cl−4MC^−^)_2_ (*N3*-binding)	19	0
*R = NO_2_ ($\sigma_{p}=+0.78$)*			
NO2-L2	2-NO_2_-4MC thione (*N3*-H, C4=S)	12	0
NO2-L4	2-NO_2_-4MC^−^ anion (*N3*-H, *N1*^−^)	11	−1
NO2-L6	(2-NO_2_-4MC-*N3*H)_2_ disulfide	22	0
NO2-C2	Cu(I)(2-NO_2_−4MC−N_3_H)_3_^+^ (S-monodentate)	37	+1
NO2-D4	*cis*-Cu(II)(2-NO_2_−4MC^−^)_2_ (*N3*-binding)	23	0

**Table 2:** Tautomer free energies in aqueous solution.

Tautomer	$G_{\mathrm{soln}}^{\circ }$ (Ha)	$G_{\mathrm{soln}}^{\circ }$ (kJ mol^−1^)	ΔG (kJ mol^−1^)
4MC thiol (L1)	−624.509480	−1,639,649.39	0.00
4MC thione (L2)	−624.510455	−1,639,651.95	−2.56

**Table 3:** Cu(II) isomer relative energies and Boltzmann populations at 298.15 K.

Isomer	$G^{\circ }$ (kJ mol^−1^)	ΔG (kJ mol^−1^)	Population (%)
D1: *trans*-(*N1*)	−7,584,199.70	29.48	0.00
D2: *cis*-(*N1*)	−7,584,195.13	34.05	0.00
D3: *trans*-(*N3*)	−7,584,225.58	3.60	19.0
D4: *cis*-(*N3*)	−7,584,229.18	0.00	81.0

**Table 4:** Calculated reduction potentials.

Redox Couple	$\Delta_{r}G^{\circ }$ (kJ mol^−1^)	$E^{\circ }$ (V)
Equation (1): (4MC)_2_/4MC^t^	−55.17	**0.286**
Equation (2): Cu(II)/Cu(I) (D1, *trans*-*N1*)	−217.29	1.126
Equation (2): Cu(II)/Cu(I) (D2, *cis*-*N1*)	−226.44	1.173
Equation (2): Cu(II)/Cu(I) (D3, *trans*-*N3*)	−165.52	0.858
Equation (2): Cu(II)/Cu(I) (D4, *cis*-*N3*)	−158.33	0.821
Equation (2): Cu(II)/Cu(I) (Boltzmann avg.)	—	0.828
EMF	—	+0.542

**Table 5:** Selected structural parameters for Cu(I) and Cu(II) complexes (bond lengths in Å, angles in degrees).

Species	Cu–S1	Cu–S2	Cu–N	S–Cu–S	N–Cu–N
C1: Cu(I)(4MC)_3_^+^	2.353	2.303	1.960	105.8–110.8 ^*a*^	16.9
C2: Cu(I)(4MC-N_3_H)_3_^+^	2.249	2.326	—	112.2–126.6 ^*a*^	25.9
D1: *trans*-Cu(II)(4MC^−^)_2_ (*N1*)	2.224	2.224	—	180.0	36.7
D2: *cis*-Cu(II)(4MC^−^)_2_ (*N1*)	2.153	5.776	1.891	171.2	14.7
D3: *trans*-Cu(II)(4MC^−^)_2_ (*N3*)	2.159	2.159	—	180.0	28.0
D4: *cis*-Cu(II)(4MC^−^)_2_ (*N3*)	2.189	2.394	2.898; 2.009 ^*b*^	129.8	14.5

^*a*^ Three S–Cu–S angles for the tricoordinate Cu(I) complexes. ^*b*^ Two Cu–N distances: Cu–N1 = 2.898 Å (weakly bound), Cu–N2 = 2.009 Å (strongly bound).

**Table 6:** Comparison of reduction potentials and EMF with literature values. All potentials in V vs. SHE.

System	$E_{\mathrm{disulfide}/\mathrm{thiol}}^{\circ }$	$E_{\begin{array}{c} \mathrm{Cu}(\mathrm{II})/\mathrm{Cu}(I) \end{array}}^{\circ }$	EMF
4MC (this study)	0.286	0.828	0.542
OSH [10]	0.310	1.320	1.020
ESH [10]	0.700	1.390	0.690
OSeH [12]	0.250	1.150	0.900
ESeH [12]	0.570	1.240	0.670
Aqueous Cu(II)/Cu(I) ^*a*^	—	0.160	—

^*a*^ Experimental value for the bare aqueous Cu(II)/Cu(I) couple.

**Table 7:** Functional benchmark: reduction potentials (V vs. SHE) and EMF from single-point energies at BP86/def2-TZVP/IEFPCM and M06-2X/def2-TZVP/IEFPCM on the same BP86/def2-SVP geometries, with BP86/def2-SVP thermal corrections (Equation (1)).

Level	$E_{\mathrm{disulfide}/\mathrm{thiol}}^{\circ }$	$E_{\begin{array}{c} \mathrm{Cu}(\mathrm{II})/\mathrm{Cu}(I) \end{array}}^{\circ }$ ^*a*^	EMF
BP86/def2-TZVP/IEFPCM	0.286	0.828	0.542
M06-2X/def2-TZVP/IEFPCM	0.160	1.063	0.904
$\Delta E^{\circ }$ (M06-2X − BP86)	−0.126	+0.236	+0.362

^*a*^ Boltzmann-weighted average over the four Cu(II) isomers; the population ordering (D4 dominant) is unchanged at both levels.

**Table 8:** Substituent effects on reduction potentials (solution-phase values at BP86/def2-TZVP/IEFPCM(water)). $\sigma_{p}$ values from [39].

Substituent	$\sigma_{p}$	$E_{\mathrm{dis}}^{\circ }$ (V)	$E_{\mathrm{Cu}}^{\circ }$ (V)	EMF (V)
CH_3_	−0.17	0.315	0.900	+0.586
H	+0.00	0.286	0.821	+0.535
Cl	+0.23	0.109	0.411	+0.302
NO_2_	+0.78	0.272	1.009	+0.738

**Table 9:** Hammett regression parameters ($E^{\circ }=\rho \;\sigma_{p}+E_{0}^{\circ }$).

Redox Couple	ρ (V)	$E_{0}^{\circ }$ (V)	$R^{2}$
Disulfide/thiol	−0.044	0.254	0.039
Cu(II)/Cu(I)	+0.134	0.757	0.045
EMF	+0.178	0.502	0.167

**Table 10:** Reaction free energies ($\Delta G^{\circ }$, kJ mol^−1^) for the disulfide/thiol and Cu(II)/Cu(I) half-reactions, and the overall EMF. Solution-phase values at BP86/def2-TZVP/IEFPCM(water).

Substituent	$\sigma_{p}$	$\Delta G_{\mathrm{dis}}^{\circ }$	$\Delta G_{\mathrm{Cu}}^{\circ }$	EMF (V)
CH_3_	−0.17	−60.7	−173.7	+0.586
H	+0.00	−55.2	−158.3	+0.535
Cl	+0.23	−21.0	−79.3	+0.302
NO_2_	+0.78	−52.4	−194.7	+0.738

**Table 11:** Cu–S and Cu–N bond lengths (Å) for derivative copper complexes (BP86/def2-SVP optimised). The Cu(I) complexes are three-coordinate trigonal planar; the Cu(II) complexes are the D4 *cis*-*N3* isomer.

*Cu(I) complexes*				
Substituent	Cu–S1	Cu–S2	Cu–S3	〈Cu–S〉
H (C2)	2.249	2.326	2.302	2.292
CH_3_	2.324	2.309	2.250	2.294
Cl	2.249	2.327	2.298	2.291
NO_2_	2.312	2.286	2.253	2.284
*Cu(II) complexes (D4 isomer)*				
Substituent	Cu–S1	Cu–S2	Cu–N ^*a*^	
H (D4)	2.189	2.394	—	
CH_3_	2.164	2.166	—	
Cl	2.164	2.169	—	
NO_2_	2.162	6.318 ^*b*^	2.766	

^*a*^ Cu–N distance when a Cu–N bond is present. ^*b*^ Cu–S2 is effectively dissociated (see text).

**Table 12:** Frontier orbital energies and HOMO–LUMO gaps for key species (BP86/def2-TZVP/IEFPCM). All energies in eV.

Species	${\mathrm{HOMO}}_{\alpha }$	${\mathrm{LUMO}}_{\alpha }$	${\mathrm{Gap}}_{\alpha }$	${\mathrm{HOMO}}_{\beta }$	${\mathrm{LUMO}}_{\beta }$	${\mathrm{Gap}}_{\beta }$
L2 (thione)	−4.62	−1.58	3.04	—	—	—
L4 (anion)	−3.86	−0.04	3.83	—	—	—
C2 (Cu(I))	−4.32	−1.83	2.49	−4.32	−1.83	2.49
D4 (Cu(II))	−4.88	−1.16	3.71	−4.83	−4.52	0.31
NO_2_–Cu(II)	−5.80	−4.24	1.56	−5.66	−5.48	0.18

**Table 13:** HOMO–LUMO gaps (eV) for all parent and derivative species. Closed-shell species show a single gap; open-shell Cu(II) species show α and β gaps. BP86/def2-TZVP/IEFPCM.

Species	H (Parent)	CH_3_	Cl	NO_2_
Thione	3.04	3.09	2.89	1.32
Anion	3.83	3.82	3.48	1.64
Disulfide	2.81	2.63	2.66	2.28
Cu(I)	2.49	2.60	2.36	0.77
Cu(II) α	3.71	3.90	3.84	1.56
Cu(II) β	0.31	0.35	0.37	0.18

**Table 14:** NPA charges on Cu and coordinated S atoms from NBO analysis.

Species	Cu NPA	S Atoms (NPA)	Avg. Cu–S WBI
L2 (free thione)	—	S6: −0.437	—
C2 (Cu(I))	+0.294	S7: −0.203; S17: −0.194; S27: −0.205	0.071
D4 (Cu(II))	+0.532	S7: +0.072; S16: −0.074	0.143
NO_2_–Cu(II)	+0.446	S7: +0.039; S18: +0.121	0.083
Cl-C2 (Cu(I))	+0.297	S7: −0.195; S17: −0.186; S27: −0.197	0.071
Cl-D4 (Cu(II))	+0.340	S7: +0.036; S16: +0.009	0.137

**Table 15:** Wiberg bond indices for Cu–S and S–S bonds. NBO analysis with IEFPCM for the copper complexes; gas phase for the two disulfides (see Section 2.1).

Species	Bond	WBI
C2 (Cu(I))	Cu1–S7	0.078
	Cu1–S17	0.069
	Cu1–S27	0.066
D4 (Cu(II))	Cu1–S7	0.185
	Cu1–S16	0.101
NO_2_–Cu(II)	Cu1–S7	0.148
	Cu1–S18	0.017
Cl-C2 (Cu(I))	Cu1–S7	0.078
	Cu1–S17	0.069
	Cu1–S27	0.067
Cl-D4 (Cu(II))	Cu1–S7	0.139
	Cu1–S16	0.134
L6 (disulfide)	S6–S15	0.221
Cl-L6 (Cl disulfide)	S6–S15	0.217

**Table 16:** Mulliken spin densities (>|0.01|) for open-shell Cu(II) species.

Atom	D4 (Cu(II))	NO_2_–Cu(II)
Cu1	0.256	0.120
S7	0.347	0.236
S16/S18	0.207 (S16)	0.298 (S18)
C6	0.120	0.077
C3	0.041	0.065
N13	0.035	−0.025
C14	—	0.092
C17	—	0.101

**Table 17:** QTAIM bond critical point properties for Cu–S bonds: electron density ρ(r), Laplacian $\nabla^{2}\rho \left(r\right)$, electron localisation function (ELF), and energy density H(r) (all in a.u.).

Species	Bond	ρ(r)	$\nabla^{2}\rho \left(r\right)$	ELF	H(r)
C2 (Cu(I))	Cu1–S7	0.078	0.206	0.228	−0.024
	Cu1–S17	0.068	0.177	0.214	−0.017
	Cu1–S27	0.070	0.188	0.215	−0.019
D4 (Cu(II))	Cu1–S7	0.091	0.207	0.279	−0.033
	Cu1–S16	0.060	0.131	0.251	−0.014
NO_2_–Cu(II)	Cu1–S7	0.096	0.212	0.289	−0.037

## Data Availability

The datasets generated and/or analyzed during the current study are available from the corresponding author on reasonable request.

## Supplementary Materials

The following supporting information can be downloaded at https://www.mdpi.com/article/10.3390/molecules31183331/s1. Reference [39] is cited in the Supplementary Materials.
