# Supplementary material for: DFT Investigation of 4-Mercaptoimidazole–Copper(I/II) Redox Chemistry and C2-Substituent Effects: A Minimal Scaffold Study of Ovothiol-Type Redox Cycling
Source: Molecules. 2026 Sep 19;31(18):3331. doi: 10.3390/molecules31183331 (PMC13609627; doi:10.3390/molecules31183331)
Supplement: Supplementary file 1 [file molecules-31-03331-s001.zip › molecules-4562724-supplementary.pdf]

Supporting Information: DFT Investigation of 4-Mercaptoimidazole–Copper(I/II) Redox Chemistry and C2-Substituent Effects: A Minimal Scaffold Study of Ovothiol-Type Redox Cycling

Do Tuong Ha[†] and Thuat T. Trinh[*,‡]

†*Faculty of Applied Sciences, Ton Duc Thang University, Ho Chi Minh City, Vietnam*

‡*Porelab, Department of Chemistry, Norwegian University of Science and Technology, NTNU, Trondheim, NO-7491, Norway*

E-mail: thuat.trinh@ntnu.no

This Supporting Information provides detailed computational data underpinning the results reported in the main text. Tables S1–S4 collect the optimisation and single-point energies, the step-by-step reduction-potential calculations for both redox couples, and the lowest real harmonic frequencies for all twelve parent species. Tables S5–S7 report the basis-set validation (def2-SVP vs. def2-TZVP vs. def2-TZVPD). Table S8 collects the solution-phase free energies and reduction potentials for the C2-substituted derivatives, and Table S9 the derivative frontier orbital energies. Tables S10–S12 provide extended electronic structure data (complete Wiberg bond indices, Mulliken spin densities, and QTAIM bond critical

point properties) for the copper complexes analysed in Section 3.9 of the main text. Finally, Table S13 reports the Cartesian coordinates of all 27 BP86/def2-SVP gas-phase optimised geometries.

Energies for All 12 Species

Table S1 lists the BP86/def2-SVP optimisation energies (E_{opt}), the BP86/def2-TZVP single-point IEFPCM energies (E_{SP}), the thermal corrections to the Gibbs free energy (ΔG_{corr}), the resulting solution Gibbs free energies ($G^{\circ}_{\mathrm{soln}}$), and the expectation value $\langle S^2 \rangle$ for all 12 species. All energies are in Hartree.

Table S1: Optimisation energies, single-point IEFPCM energies, thermal corrections, solution Gibbs free energies, and $\langle S^2 \rangle$ for all 12 species. All energies in Hartree.

Species	E_{opt}	E_{SP}	ΔG_{corr}	$G^{\circ}_{\mathrm{soln}}$	$\langle S^2 \rangle$
L1_4MC	−624.15156	−624.54789	0.03841	−624.50948	—
L2_4MC_N3H	−624.14008	−624.55240	0.04194	−624.51046	—
L3_anion_N1H	−623.57231	−624.08090	0.02803	−624.05287	—
L4_anion_N3H	−623.59652	−624.08770	0.02926	−624.05844	—
L5_disulfide_4MC	−1247.11453	−1247.90669	0.08202	−1247.82467	0.000
L6_disulfide_N3H	−1247.11976	−1247.90768	0.08295	−1247.82473	0.000
C1_Cu1_4MC3	−3512.91154	−3514.36935	0.15933	−3514.21001	0.000
C2_Cu1_4MCN3H3	−3512.92310	−3514.40433	0.16758	−3514.23675	0.000
D1_Cu2_trans_N1	−2887.71482	−2888.74748	0.07833	−2888.66915	0.7532
D2_Cu2_cis_N1	−2887.71448	−2888.75510	0.08769	−2888.66741	0.7534
D3_Cu2_trans_N3	−2887.71212	−2888.75780	0.07879	−2888.67901	0.7523
D4_Cu2_cis_N3	−2887.72322	−2888.76016	0.07978	−2888.68038	0.7523

Equation 1 Calculation (Disulfide/Thiol Couple)

Table S2 details the thermodynamic quantities used to compute the reduction potential of the disulfide/thiol couple (Equation 1 in the main text).

Table S2: Thermodynamic quantities for Equation 1 (disulfide/thiol couple).

Quantity	Value
$G^\circ[\mathrm{4MC^t}]$ (thione, L2)	-624.51046 Ha = $-1\,639\,651.95$ kJ mol^{-1}
$G^\circ[\mathrm{(4MC)_2}]$ (thione disulfide, L6)	-1247.82473 Ha = $-3\,276\,700.51$ kJ mol^{-1}
$\mu[\mathrm{H^+(aq)}]$	-1124.2 kJ mol^{-1}
$\mu[e^-_{\mathrm{SHE}}]$	-418.5 kJ mol^{-1}
$\Delta_r G^\circ(\mathrm{eq}\,1)$	-55.17 kJ mol^{-1}
$E^\circ(\mathrm{eq}\,1)$	**0.2859 V**

Equation 2 Calculation (Cu(II)/Cu(I) Couple)

Table S3 lists the Gibbs free energies of the four Cu(II) isomers and the dominant Cu(I) complex, the reaction free energies, and the individual and Boltzmann-averaged reduction potentials for Equation 2 in the main text.

Table S3: Equation 2 calculation: Cu(II)/Cu(I) reduction potentials for each isomer and the Boltzmann-weighted average.

Isomer	G°[Cu(II)] (kJ mol^{-1})	G°[Cu(I)] (kJ mol^{-1})	$\Delta_r G^\circ$ (kJ mol^{-1})	E° (V)
D1: *trans*-(*N1*)	$-7\,584\,199.70$	$-9\,231\,239.71$	-217.29	1.1261
D2: *cis*-(*N1*)	$-7\,584\,195.13$	$-9\,231\,239.71$	-226.44	1.1734
D3: *trans*-(*N3*)	$-7\,584\,225.58$	$-9\,231\,239.71$	-165.52	0.8578
D4: *cis*-(*N3*)	$-7\,584\,229.18$	$-9\,231\,239.71$	-158.33	0.8205
**Boltzmann avg**	—	—	—	**0.8276**

G°[Cu(I) complex C2] = -3514.23675 Ha = $-9\,231\,239.71$ kJ mol^{-1}.

Lowest Real Harmonic Frequencies

Table S4 lists the five lowest real harmonic frequencies for each species. All species have zero imaginary frequencies, confirming true minima on the potential energy surface.

Table S4: Five lowest real harmonic frequencies (cm^{-1}) for each species. All species have zero imaginary frequencies.

Species	ν_1	ν_2	ν_3	ν_4	ν_5
L1_4MC	215.21	257.72	286.15	455.86	540.43
L2_4MC_N3H	215.83	302.10	506.10	570.32	595.83
L3_anion_N1H	221.68	313.43	314.21	480.03	610.68
L4_anion_N3H	225.14	297.43	483.14	535.03	645.76
L5_disulfide_4MC	35.15	69.84	82.35	109.67	122.01
L6_disulfide_N3H	46.38	79.93	101.03	115.52	121.61
C1_Cu1_4MC3	39.60	47.72	56.49	66.09	72.47
C2_Cu1_4MCN3H3	30.87	34.11	45.43	58.04	77.81
D1_Cu2_trans_N1	43.12	43.23	54.03	83.96	94.38
D2_Cu2_cis_N1	45.49	91.40	133.30	151.95	163.60
D3_Cu2_trans_N3	35.69	50.33	67.82	77.49	83.96
D4_Cu2_cis_N3	50.43	51.87	74.27	78.62	105.82

Basis-Set Validation: def2-SVP vs def2-TZVP vs def2-TZVPD

To validate the use of def2-SVP for geometry optimisations reported in the main text, we re-optimised two representative species—the free ligand 4MC (L1, organic scaffold) and the dominant Cu(I) complex C2—with the larger def2-TZVP and def2-TZVPD basis sets at the same BP86/IEFPCM(water) level of theory. All optimisations employed the RI-J approximation and Cartesian coordinates.

Tables S5 and S6 compare key bond lengths and angles across the three basis sets, while Table S7 reports the heavy-atom root-mean-square deviation (RMSD) after Kabsch alignment.

L1 (4MC)

For the free ligand, all three basis sets yield virtually identical geometries. The maximum bond-length deviation between def2-SVP and def2-TZVP is 0.010 Å (C=S thione), and the maximum angle deviation is 0.22°. The heavy-atom RMSD between SVP and TZVP is only 0.006 Å, confirming that def2-SVP provides quantitatively reliable geometries for this organic scaffold.

Table S5: Key bond lengths (Å) and angles (°) for L1 (4MC) optimised at BP86/IEFPCM with three basis sets.

Parameter	def2-SVP	def2-TZVP	def2-TZVPD	Δ(SVP–TZVP)
Bond lengths (Å)				
C=S (thione)	1.7752	1.7652	1.7651	+0.0100
C2–N3	1.3272	1.3246	1.3244	+0.0027
C4–N1	2.1999	2.1927	2.1925	+0.0073
C5–N1	1.3883	1.3857	1.3855	+0.0026
C2–N1	1.3695	1.3647	1.3651	+0.0048
C5–C4	1.3937	1.3834	1.3832	+0.0104
N3–C4	1.3776	1.3790	1.3792	−0.0014
Bond angles (°)				
N1–C2–N3	111.65	111.54	111.49	+0.11
C2–N3–C4	105.44	105.41	105.44	+0.04
N3–C4–C5	110.78	110.70	110.68	+0.08
C4–C5–N1	104.52	104.72	104.73	−0.20
C5–N1–C2	107.61	107.64	107.65	−0.02
S–C4–N3	122.17	122.23	122.29	−0.06
S–C4–C5	127.05	127.07	127.03	−0.02

C2 (Cu(I) complex)

For the Cu(I) complex, the Cu–S coordination bonds and S–C thione bonds are consistent across all three basis sets. The Cu–S distances vary by at most 0.04 Å between SVP and TZVP, and the S–C thione bonds agree within 0.01 Å. The sum of S–Cu–S angles remains $\approx$ 360° (trigonal planar) in all cases. The larger full-molecule heavy-atom RMSD (see Table S7) arises from rotational flexibility of the three 4MC ligands about the Cu–S bonds

(soft modes with harmonic frequencies < 40 cm^{-1}, cf. Table S4), not from differences in the coordination sphere.

Table S6: Key bond lengths (Å) and angles (°) for C2 ([Cu(4 MC−N_3H)$_3$]) optimised at BP86/IEFPCM with three basis sets.

Parameter	def2-SVP	def2-TZVP	def2-TZVPD	Δ(SVP–TZVP)
Bond lengths (Å)				
Cu–S1	2.2486	2.2362	2.2800	+0.0125
Cu–S2	2.3256	2.2820	2.3058	+0.0436
Cu–S3	2.3019	2.3096	2.2955	−0.0077
S1–C (thione)	1.7190	1.7135	1.7084	+0.0055
S2–C (thione)	1.7249	1.7136	1.7140	+0.0113
S3–C (thione)	1.7212	1.7119	1.7056	+0.0093
Bond angles (°)				
S1–Cu–S2	126.64	128.71	124.49	−2.07
S1–Cu–S3	112.18	110.40	110.90	+1.78
S2–Cu–S3	120.97	120.84	123.00	+0.13
Σ(S–Cu–S)	359.79	359.95	358.38	−0.16

Table S7: Heavy-atom RMSD and maximum atomic displacement (Å) after Kabsch alignment of optimised geometries.

Species	SVP vs TZVP	SVP vs TZVPD	TZVP vs TZVPD
L1 (4MC)	0.006 / 0.011	0.007 / 0.011	0.001 / 0.001
C2 (Cu(I))	0.447 / 0.771	1.132 / 1.744	0.884 / 1.764

For L1, the negligible RMSD (≤ 0.007 Å) confirms that def2-SVP geometries are quantitatively indistinguishable from triple-ζ results. For C2, the coordination bonds (Cu–S, S–C) agree within 0.04 Å, but the full-molecule RMSD is larger because the three 4MC ligands can rotate about the Cu–S bonds with very low energy barriers. These soft torsional modes do not affect the thermodynamic properties computed in the main text (energies are evaluated with def2-TZVP single-point calculations on def2-SVP geometries), confirming that the two-step protocol is well-converged.

C2-Substituted Derivatives: Solution-Phase Free Energies and Reduction Potentials

Three C2-substituted derivatives of 4MC were studied to probe the electronic substituent effect on the redox chemistry: **-CH$_3$** ($\sigma_p = -0.17$), **-Cl** ($\sigma_p = +0.23$), and **-NO$_2$** ($\sigma_p = +0.78$).[S1] For each substituent, five species were computed at the same level of theory as the parent (BP86/def2-SVP optimisation and frequency analysis, def2-TZVP single-point with IEFPCM water): the thione tautomer, the N3-H thione anion, the homodimeric disulfide, the Cu(I) complex, and the dominant D4 *cis-N3* Cu(II) isomer. The three derivative disulfides were re-optimised from bonded starting structures derived from the parent L6 framework; the corrected geometries feature S–S bonds of 2.18–2.22 Å and are verified by harmonic frequency analysis (zero imaginary frequencies for Cl and NO$_2$; a single -40.3 cm^{-1} flat ring-libration mode for CH$_3$, accepted as a quasi-harmonic saddle with negligible thermochemical impact).

Table S8: Solution-phase free energies (G°_{soln}, Hartree) and reduction potentials for C2-substituted derivatives.

Substituent	Species	G°_{soln}	ΔG (kJ/mol)	E° (V)
CH3	thione	-663.819842	—	—
CH3	disulfide	-1326.441396	-60.70	0.3146
CH3	Cu(I)	-3632.166806	—	—
CH3	Cu(II)	-2967.298115	-173.71	0.9002
CH3	EMF	—	—	+0.5856
H	thione	-624.510455	—	—
H	disulfide	-1247.824732	-55.17	0.2859
H	Cu(I)	-3514.236755	—	—
H	Cu(II)	-2888.680379	-158.33	0.8205
H	EMF	—	—	0.5346

Table S8: (continued)

Substituent	Species	G°_{soln}	ΔG (kJ/mol)	E° (V)
Cl	thione	-1084.158061	—	—
Cl	disulfide	-2167.132943	-21.03	0.1090
Cl	Cu(I)	-4893.178000	—	—
Cl	Cu(II)	-3807.989074	-79.27	0.4108
Cl	EMF	—	—	+0.3018
NO2	thione	-829.121182	—	—
NO2	disulfide	-1657.047237	-52.41	0.2716
NO2	Cu(I)	-4128.053432	—	—
NO2	Cu(II)	-3297.879396	-194.74	1.0092
NO2	EMF	—	—	+0.7376

Hammett Plot

Figure S1 shows the substituent dependence of the two reduction potentials and of the EMF as a Hammett plot (E° vs. σ_p). Both redox couples display only weak linear correlations with the substituent constant (the fitted parameters ρ, E°_0, and R^2 are summarised in Section 3.8 of the main text), and the EMF remains positive for all four substituents.

Derivative Frontier Orbital Energies

Table S9 collects the HOMO and LUMO energies for all 15 derivative species (5 species $\times$ 3 substituents), together with the parent values.

The NO_2 substituent consistently produces the smallest HOMO–LUMO gaps across all species categories, primarily by lowering the LUMO energy through the introduction of low-lying π^* orbitals on the nitro group. For the disulfide species, the parent disulfide L6 has a

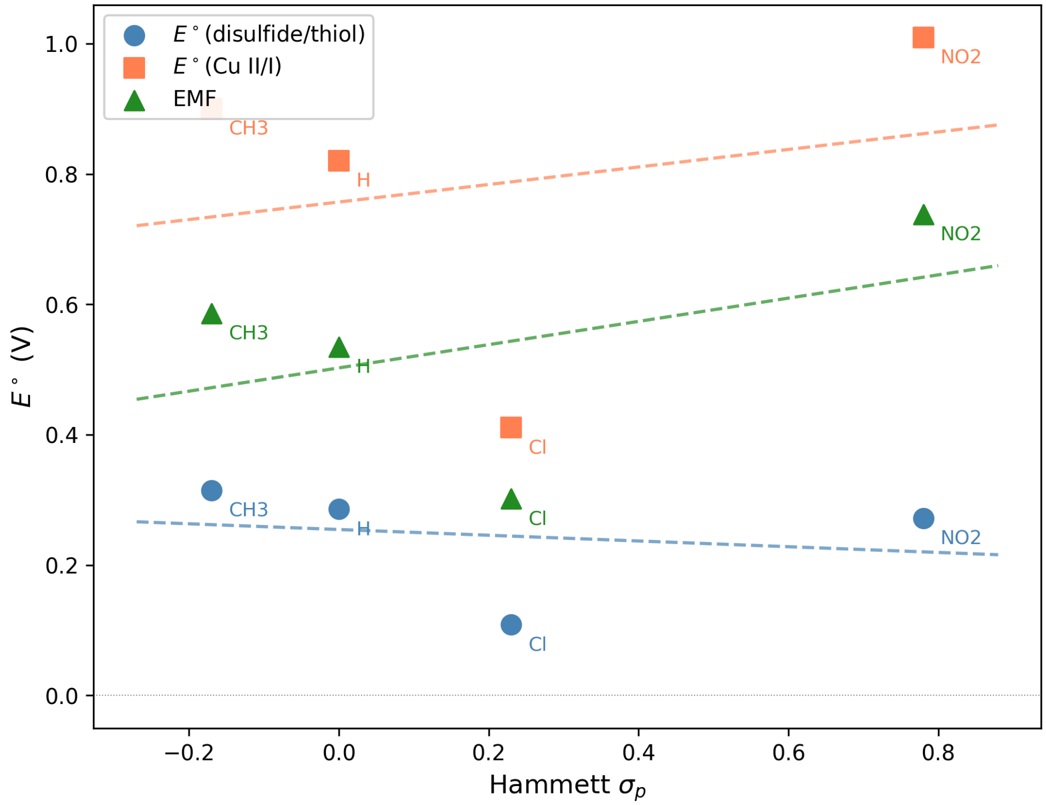

Figure S1: Hammett plot showing the linear relationship between the substituent constant σ_p and the calculated reduction potentials for the disulfide/thiol couple (E°_{dis}), the Cu(II)/Cu(I) couple (E°_{Cu}), and the EMF (solution-phase values at BP86/def2-TZVP/IEFPCM(water)). Dashed lines are linear regression fits ($E^\circ = \rho\,\sigma_p + E^\circ_0$); the fitted parameters are summarised in Section 3.8 of the main text.

gap of 2.81 eV, and the three C2-substituted disulfides show only modest changes (2.63, 2.66, and 2.28 eV for CH_3, Cl, and NO_2, respectively), mirroring the near-zero Hammett sensitivity of the disulfide/thiol couple reported in the main text (Section 3.8): the frontier orbitals of the C4–S–S–C4 redox centre are largely decoupled from the C2 substituent position.

Electronic Structure: Extended Wiberg, Spin Density, and QTAIM Data

Tables S10–S12 provide extended electronic structure data for the copper complexes analysed in Section 3.9 of the main text: the dominant Cu(I) complex C2, the dominant Cu(II)

Table S9: Frontier orbital energies and HOMO–LUMO gaps for all parent and derivative species (BP86/def2-TZVP/IEFPCM). All energies in eV (converted from Hartree by ×27.2114). Closed-shell species: single α gap. Open-shell Cu(II): α and β gaps.

Substituent	Species	$HOMO_\alpha$	$LUMO_\alpha$	Gap_α	Gap_β
H	Thione (L2)	−4.62	−1.58	3.04	—
H	Anion (L4)	−3.86	−0.04	3.83	—
H	Disulfide (L6)	−5.80	−2.99	2.81	—
H	Cu(I) (C2)	−4.32	−1.83	2.49	—
H	Cu(II) (D4)	−4.88	−1.16	3.71	0.31
CH3	Thione	−4.50	−1.41	3.09	—
CH3	Anion	−3.73	+0.09	3.82	—
CH3	Disulfide	−5.56	−2.93	2.63	—
CH3	Cu(I)	−4.22	−1.62	2.60	—
CH3	Cu(II)	−4.98	−1.08	3.90	0.35
Cl	Thione	−4.68	−1.79	2.89	—
Cl	Anion	−3.97	−0.49	3.48	—
Cl	Disulfide	−5.80	−3.14	2.66	—
Cl	Cu(I)	−4.42	−2.06	2.36	—
Cl	Cu(II)	−5.19	−1.35	3.84	0.37
NO2	Thione	−5.40	−4.08	1.32	—
NO2	Anion	−4.68	−3.04	1.64	—
NO2	Disulfide	−6.50	−4.22	2.28	—
NO2	Cu(I)	−5.09	−4.31	0.77	—
NO2	Cu(II)	−5.80	−4.24	1.56	0.18

isomer D4, and the NO_2-substituted Cu(II) complex. All calculations were performed at the BP86/def2-TZVP/IEFPCM(water) level with `GUI=2` and `NBO=1` keywords. The frontier orbital energies and NPA charges for the five representative species are collected in the main text; the tables below complement these summaries with the complete Wiberg bond indices including the total Cu valence, the full Mulliken spin populations for the open-shell Cu(II) species, and the QTAIM bond critical point properties including the kinetic and potential energy densities $G(r)$ and $V(r)$.

Table S10: Wiberg bond indices (WBI) for Cu–S bonds and total bond indices per atom.

Species	Bond	WBI	Cu Total WBI
C2 (Cu(I))	Cu1–S7	0.078	0.214
	Cu1–S17	0.069	
	Cu1–S27	0.066	
D4 (Cu(II))	Cu1–S7	0.185	0.286
	Cu1–S16	0.101	
NO_2–Cu(II)	Cu1–S7	0.148	0.165
	Cu1–S18	0.017	

Table S11: Mulliken spin densities for all atoms with |spin| > 0.01 in the open-shell Cu(II) species.

Atom	D4 (Cu(II))	NO_2–Cu(II)
Cu1	+0.256	+0.120
S7	+0.347	+0.236
S16/S18	+0.207	+0.298
C3	+0.041	+0.065
C6	+0.120	+0.077
N13	+0.035	−0.025
C14	—	+0.092
N15	—	+0.011
C16	—	−0.023
C17	—	+0.101
N2/N21	—	−0.012
O11/O22	—	+0.011/+0.045
O12/O23	—	+0.015/+0.012

Table S12: QTAIM properties at Cu–S bond critical points: electron density $\rho(r)$, Laplacian $\nabla^2\rho(r)$, ELF, energy density $H(r)$, kinetic energy $G(r)$, and potential energy $V(r)$ (all in a.u.).

Species	Bond	ρ	$\nabla^2\rho$	ELF	$H(r)$	$G(r)$	$V(r)$
C2 (Cu(I))	Cu1–S7	0.078	+0.206	0.228	−0.024	0.075	−0.099
	Cu1–S17	0.068	+0.177	0.214	−0.017	0.062	−0.079
	Cu1–S27	0.070	+0.188	0.215	−0.019	0.066	−0.085
D4 (Cu(II))	Cu1–S7	0.091	+0.207	0.279	−0.033	0.085	−0.118
	Cu1–S16	0.060	+0.131	0.251	−0.014	0.046	−0.060
NO_2–Cu(II)	Cu1–S7	0.096	+0.212	0.289	−0.037	0.090	−0.127

Optimised Cartesian Coordinates

Table S13 reports the Cartesian coordinates of the BP86/def2-SVP gas-phase optimised geometries for all 27 species catalogued in Table 1 of the main text. The geometries are those used for the frequency analyses and single-point energy calculations of the main text. Two documented features are visible in the coordinates: the CH_3 disulfide retains the single small (-40.3 cm^{-1}) flat ring-libration mode discussed in Section , and the NO_2-substituted Cu(II) complex retains the dissociated Cu–S contact (6.32 Å) discussed in Section 3.4 of the main text.

Table S13: Cartesian coordinates (Å) of the BP86/def2-SVP gas-phase optimised geometries of all 27 species (atom ordering follows the Q-Chem input of each optimisation).

Atom	x	y	z
**L1 — 4MC thiol (*N1*–H, C4–SH)**			
N	-0.8774	0.6405	0.0000
C	-0.6282	-0.7061	0.0000
N	0.6739	-0.9631	0.0000
C	1.2909	0.2686	0.0000
C	0.3473	1.2943	0.0000
S	3.0578	0.4406	0.0000
H	3.2589	-0.9065	0.0000
H	-1.7948	1.0892	0.0000
H	-1.4302	-1.4564	0.0000
H	0.4341	2.3853	0.0000
**L2 — 4MC thione (*N3*–H, C4=S)**			
N	-0.9383	0.6634	0.0000
C	-0.9556	-0.6832	0.0000
N	0.3406	-1.0776	0.0000

Table S13 (continued)

Atom	x	y	z
C	1.2498	0.0285	0.0000
C	0.3637	1.1385	0.0000
S	2.9224	-0.1330	0.0000
H	0.6826	-2.0417	0.0000
H	-1.8457	-1.3187	0.0000
H	0.5950	2.2063	0.0000
H	-1.7796	1.2431	0.0000
**L3 — 4MC^{-} thiolate (*N1*–H, S^{-})**			
N	-0.9480	0.6863	0.0000
C	-0.8924	-0.6865	0.0000
N	0.3500	-1.1413	0.0000
C	1.2087	-0.0075	0.0000
C	0.3782	1.1381	0.0000
S	2.9389	-0.0737	0.0000
H	-1.7886	1.2653	0.0000
H	-1.8103	-1.2998	0.0000
H	0.6290	2.2052	0.0000
**L4 — 4MC^{-} thione anion (*N3*–H, *N1*$^{-}$)**			
N	-1.0043	0.6889	0.0000
C	-0.9519	-0.6377	0.0000
N	0.3581	-1.0826	0.0000
C	1.2310	0.0211	0.0000
C	0.3169	1.1044	0.0000
S	2.9493	-0.1489	0.0000

Table S13 (continued)

Atom	x	y	z
H	0.7147	-2.0396	0.0000
H	-1.8139	-1.3241	0.0000
H	0.5881	2.1702	0.0000
**L5 — $(4MC)_2$ disulfide (thiol, *N1*–H)**			
N	0.3313	0.8011	0.2628
C	1.3171	0.5908	1.2001
N	2.4366	0.1603	0.6438
C	2.1775	0.1004	-0.7149
C	0.8678	0.5035	-0.9735
S	3.3151	-0.5043	-1.9316
H	-0.6221	1.1210	0.4457
H	1.1553	0.7704	2.2719
H	0.3054	0.5985	-1.9081
N	7.0249	-0.3267	1.0462
C	7.9252	-0.2352	0.0058
N	7.3434	0.1787	-1.1078
C	6.0191	0.3766	-0.7707
C	5.7944	0.0769	0.5728
S	4.8431	0.9787	-1.9571
H	7.2240	-0.6465	1.9962
H	8.9883	-0.4852	0.1256
H	4.8676	0.0814	1.1607
**L6 — $(4MC\text{-}N3H)_2$ disulfide (thione, *N3*–H)**			
N	0.2115	0.6896	0.3939

Table S13 (continued)

Atom	x	y	z
C	0.9829	-0.1003	1.1399
N	2.0862	-0.5280	0.4589
C	2.0316	0.0346	-0.8176
C	0.8484	0.7872	-0.8127
S	3.1995	-0.2851	-2.0677
H	0.7840	-0.3895	2.1814
H	0.4463	1.3791	-1.6458
H	2.8392	-1.1318	0.7981
N	6.8195	-0.5741	1.4421
C	7.3375	-1.1819	0.3767
N	6.7719	-0.7420	-0.7870
C	5.8158	0.2167	-0.4527
C	5.8792	0.2904	0.9463
S	4.8588	1.0917	-1.6204
H	8.1240	-1.9495	0.3973
H	5.2878	0.9509	1.5947
H	6.9898	-1.0355	-1.7421
**C1 — Cu(I)(4 MC)$_3$$^+$ (thiol)**			
Cu	-0.0329	-1.0830	-0.9321
N	4.6350	0.3909	1.0498
C	3.7867	1.2949	0.4701
N	2.8627	0.6738	-0.2532
C	3.1313	-0.6750	-0.1403
C	4.2389	-0.8749	0.6746

Table S13 (continued)

Atom	x	y	z
S	2.1477	-1.9651	-0.8864
H	2.5589	-1.7754	-2.1815
H	5.4286	0.6150	1.6569
H	3.8810	2.3800	0.6034
H	4.7536	-1.7855	0.9987
N	-2.2167	3.7273	-0.1768
C	-2.1809	2.7618	0.7922
N	-1.3946	1.7554	0.4282
C	-0.9028	2.0857	-0.8180
C	-1.4074	3.3186	-1.2173
S	0.1717	1.0452	-1.7873
H	1.3975	1.1589	-1.0521
H	-2.7554	4.5970	-0.1413
H	-2.7409	2.8348	1.7333
H	-1.2581	3.9108	-2.1261
N	-3.3118	-3.3995	0.1851
C	-2.4903	-2.9785	-0.8191
N	-1.5766	-2.1337	-0.3364
C	-1.8324	-1.9952	1.0269
C	-2.9198	-2.7888	1.3630
S	-0.7883	-0.9967	2.0451
H	-1.0838	0.2211	1.3894
H	-4.0861	-4.0598	0.0812
H	-2.5867	-3.3040	-1.8615

Table S13 (continued)

Atom	x	y	z
H	-3.4282	-2.9561	2.3176
**C2 — $Cu(I)(4MC\text{-}N_3H)_3^+$ (thione)**			
Cu	0.5724	-0.6762	-0.6933
N	4.8099	1.3616	0.8199
C	5.6991	1.0575	-0.1422
N	5.0257	0.3409	-1.0701
C	3.6712	0.1677	-0.7120
C	3.5609	0.8422	0.5152
S	2.5799	-0.7135	-1.7057
H	5.4227	-0.0422	-1.9331
H	6.7577	1.3341	-0.1639
H	2.6779	0.9710	1.1523
H	5.0387	1.8970	1.6609
N	-2.4632	2.8524	-0.2918
C	-2.2085	3.4627	0.8796
N	-1.3422	2.6645	1.5452
C	-1.0203	1.5156	0.7941
C	-1.7618	1.6620	-0.3880
S	0.0695	0.3022	1.3557
H	-0.9523	2.8516	2.4733
H	-2.6141	4.4210	1.2184
H	-1.8139	0.9864	-1.2527
H	-3.0806	3.2329	-1.0142
N	-3.2754	-3.2955	0.7999

Table S13 (continued)

Atom	x	y	z
C	-3.7494	-3.8914	-0.3090
N	-3.0649	-3.3626	-1.3484
C	-2.1211	-2.4062	-0.9149
C	-2.2827	-2.3827	0.4796
S	-1.0831	-1.5809	-2.0122
H	-3.1795	-3.6217	-2.3328
H	-4.5269	-4.6603	-0.3539
H	-1.7439	-1.7827	1.2231
H	-3.6018	-3.5072	1.7463
**D1 — *trans*-Cu(II)(4 MC^{-})$_2$ (*N1*-binding)**			
Cu	0.0000	0.0000	0.0000
N	2.2233	2.9460	0.3561
C	2.4105	2.6806	1.6448
N	2.3325	1.3359	1.9131
C	2.0734	0.6714	0.7146
C	2.0238	1.7312	-0.2394
S	1.8718	-1.0630	0.5573
H	2.4150	0.8676	2.8186
H	2.6078	3.4256	2.4289
H	1.8992	1.6189	-1.3254
N	-2.2233	-2.9460	-0.3561
C	-2.4105	-2.6806	-1.6448
N	-2.3325	-1.3359	-1.9131
C	-2.0734	-0.6714	-0.7146

Table S13 (continued)

Atom	x	y	z
C	-2.0238	-1.7312	0.2394
S	-1.8718	1.0630	-0.5573
H	-2.4150	-0.8676	-2.8186
H	-2.6078	-3.4256	-2.4289
H	-1.8992	-1.6189	1.3254
**D2 — *cis*-Cu(II)(4 MC$^-$)$_2$ (*N1*-binding)**			
Cu	-0.1240	0.2030	-0.8730
N	4.5231	0.4315	1.7029
C	3.5276	1.2813	1.9319
N	2.4730	1.0688	1.0746
C	2.8117	0.0126	0.2376
C	4.1049	-0.3565	0.6648
S	1.8425	-0.6557	-1.0459
H	1.5910	1.5840	1.0257
H	3.5133	2.0685	2.6985
H	4.7227	-1.1656	0.2536
N	-1.9037	0.8416	-0.8434
C	-2.8492	0.4722	-1.7347
N	-4.0129	1.0996	-1.4556
C	-3.8555	1.9329	-0.3258
C	-2.5002	1.7326	0.0232
S	-5.0954	2.8853	0.3306
H	-4.8994	1.0166	-1.9604
H	-2.7051	-0.2339	-2.5612

Table S13 (continued)

Atom	x	y	z
H	-1.9652	2.1994	0.8566
**D3 — *trans*-Cu(II)(4 MC^{-})$_2$ (*N3*-binding)**			
Cu	0.0000	0.0000	0.0000
N	2.4107	2.9515	-2.7835
C	3.3774	3.4408	-1.9286
N	3.2758	2.9194	-0.7208
C	2.1983	2.0506	-0.7764
C	1.6433	2.0588	-2.0676
S	1.7156	1.1411	0.6442
H	2.2807	3.2012	-3.7663
H	4.1282	4.1746	-2.2530
H	0.7978	1.5167	-2.5053
N	-2.4107	-2.9515	2.7835
C	-3.3774	-3.4408	1.9286
N	-3.2758	-2.9194	0.7208
C	-2.1983	-2.0506	0.7764
C	-1.6433	-2.0588	2.0676
S	-1.7156	-1.1411	-0.6442
H	-2.2807	-3.2012	3.7663
H	-4.1282	-4.1746	2.2530
H	-0.7978	-1.5167	2.5053
**D4 — *cis*-Cu(II)(4 MC^{-})$_2$ (*N3*-binding)**			
Cu	-1.0749	0.3713	-0.6661
N	3.3026	1.8362	-2.3619

Table S13 (continued)

Atom	x	y	z
C	2.7864	1.9798	-1.0943
N	1.6470	1.3238	-0.9553
C	1.3975	0.7288	-2.1848
C	2.4291	1.0417	-3.0798
S	-0.0230	-0.2504	-2.4819
H	4.1720	2.2432	-2.7123
H	3.2881	2.5761	-0.3202
H	2.5923	0.7759	-4.1290
N	-2.6551	0.3089	3.2154
C	-1.6937	-0.2974	2.4667
N	-1.7019	0.2115	1.2363
C	-2.7022	1.1853	1.1767
C	-3.3143	1.2552	2.4269
S	-2.8450	1.9581	-0.3880
H	-2.8577	0.1049	4.1950
H	-1.0259	-1.0807	2.8448
H	-4.1320	1.8706	2.8119
**CH3-L2 — 2-CH_3-4MC thione (*N3*–H, C4=S)**			
N	-0.9273	0.6699	0.0000
C	-0.9542	-0.6806	0.0000
N	0.3489	-1.0642	0.0000
C	1.2609	0.0393	0.0000
C	0.3792	1.1479	0.0000
S	2.9349	-0.1363	0.0000

Table S13 (continued)

Atom	x	y	z
H	0.6932	-2.0276	0.0000
H	0.6090	2.2159	0.0000
H	-1.7673	1.2512	0.0000
C	-2.1616	-1.5564	0.0000
H	-1.8560	-2.6200	0.0000
H	-2.7973	-1.3931	0.8976
H	-2.7973	-1.3931	-0.8976
**CH3-L4 — 2-CH_3-4MC$^-$ anion (*N3*–H, *N1*$^-$)**			
N	-0.9900	0.7043	0.0000
C	-0.9547	-0.6248	0.0000
N	0.3585	-1.0713	0.0000
C	1.2399	0.0249	0.0000
C	0.3331	1.1134	0.0000
S	2.9572	-0.1580	0.0000
H	0.7086	-2.0309	0.0000
H	0.6104	2.1776	0.0000
C	-2.1399	-1.5472	0.0000
H	-1.8191	-2.6110	0.0000
H	-2.7929	-1.3989	0.8923
H	-2.7929	-1.3989	-0.8923
**CH3-L6 — (2-CH_3-4MC-*N3*H)$_2$ disulfide**			
N	0.3035	0.4172	0.3990
C	1.1752	-0.3743	1.0360
N	2.2556	-0.6516	0.2396

Table S13 (continued)

Atom	x	y	z
C	2.0761	0.0123	-0.9772
C	0.8467	0.6687	-0.8297
S	3.1731	-0.1164	-2.3184
H	0.3458	1.2956	-1.5792
H	3.0757	-1.2167	0.4730
C	1.0176	-0.8857	2.4332
H	1.0929	-1.9927	2.4836
H	1.7869	-0.4656	3.1169
H	0.0213	-0.5822	2.8039
N	6.9167	-0.5626	1.0401
C	7.4159	-1.0913	-0.0824
N	6.7924	-0.5688	-1.1872
C	5.8309	0.3450	-0.7523
C	5.9442	0.3166	0.6441
S	4.8154	1.2806	-1.8159
H	5.3673	0.9184	1.3593
H	6.9710	-0.7914	-2.1690
C	8.5106	-2.1096	-0.1478
H	9.4107	-1.7182	-0.6690
H	8.7972	-2.3781	0.8857
H	8.1910	-3.0327	-0.6771
**CH3-C2 — Cu(I)(2-CH_3–4MC–N_3H)$_3^+$**			
Cu	0.1685	-0.6400	-1.0190
N	4.4620	0.8739	0.4855

Table S13 (continued)

Atom	x	y	z
C	5.2217	0.9383	-0.6284
N	4.3493	0.8392	-1.6643
C	3.0142	0.6978	-1.2246
C	3.1162	0.7290	0.1720
S	1.7038	0.5154	-2.3270
H	4.6040	0.8325	-2.6559
H	2.3243	0.6227	0.9230
H	4.8452	0.9125	1.4330
C	6.7026	1.0763	-0.7035
H	7.1569	0.2362	-1.2704
H	7.0014	2.0212	-1.2057
H	7.1437	1.0764	0.3116
N	-1.1071	3.8593	-0.1310
C	-1.8206	3.8344	1.0149
N	-1.6498	2.5834	1.5162
C	-0.8269	1.7899	0.6885
C	-0.4876	2.6400	-0.3693
S	-0.4328	0.1506	1.0653
H	-2.0661	2.2324	2.3828
H	0.1418	2.4276	-1.2449
H	-1.0516	4.6753	-0.7456
C	-2.6519	4.9298	1.5871
H	-2.3732	5.1413	2.6406
H	-2.5167	5.8625	1.0073

Table S13 (continued)

Atom	x	y	z
H	-3.7336	4.6735	1.5697
N	-3.4059	-3.6945	0.4315
C	-3.4793	-4.6442	-0.5246
N	-2.5279	-4.3113	-1.4340
C	-1.8275	-3.1387	-1.0703
C	-2.4188	-2.7598	0.1418
S	-0.5658	-2.4999	-2.0503
H	-2.3190	-4.8310	-2.2908
H	-2.1931	-1.8982	0.7804
H	-4.0068	-3.6790	1.2583
C	-4.4317	-5.7876	-0.5882
H	-3.9045	-6.7376	-0.8147
H	-4.9538	-5.9138	0.3799
H	-5.2037	-5.6358	-1.3743
**CH3-D4 — *cis*-Cu(II)(2-CH_3−4 MC^-)$_2$ (*N3*-binding)**			
Cu	-0.1391	0.6851	-0.5359
N	4.5429	2.5816	-2.0504
C	3.5960	3.3294	-1.4747
N	2.5040	2.5583	-1.1374
C	2.7625	1.2449	-1.5140
C	4.0538	1.3079	-2.0845
S	1.7210	-0.1239	-1.2883
H	1.6451	2.8642	-0.6720
H	4.6213	0.4676	-2.5052

Table S13 (continued)

Atom	x	y	z
C	3.6682	4.8036	-1.2448
H	3.0756	5.3656	-2.0001
H	3.2899	5.0869	-0.2404
H	4.7229	5.1238	-1.3347
N	-3.4344	-1.7920	1.7863
C	-4.5014	-0.9916	1.7139
N	-4.1643	0.2186	1.1456
C	-2.8084	0.1924	0.8326
C	-2.3926	-1.0868	1.2540
S	-2.0010	1.5683	0.1297
H	-4.7857	1.0095	0.9626
H	-1.3742	-1.4955	1.1924
C	-5.8818	-1.3327	2.1733
H	-6.6097	-1.3444	1.3331
H	-5.8590	-2.3402	2.6273
H	-6.2589	-0.6125	2.9315
**Cl-L2 — 2-Cl-4MC thione (*N3*–H, C4=S)**			
N	-0.9439	0.6625	0.0000
C	-0.9501	-0.6833	0.0000
N	0.3420	-1.0864	0.0000
C	1.2516	0.0327	0.0000
C	0.3651	1.1380	0.0000
S	2.9208	-0.1319	0.0000
H	0.6742	-2.0538	0.0000

Table S13 (continued)

Atom	x	y	z
H	0.5905	2.2070	0.0000
H	-1.7931	1.2310	0.0000
Cl	-2.3479	-1.6724	0.0000
**Cl-L4 — 2-Cl-4MC^{-} anion (*N3*–H, *N1*$^{-}$)**			
N	-1.0070	0.6930	0.0000
C	-0.9399	-0.6142	0.0000
N	0.3529	-1.0859	0.0000
C	1.2333	0.0249	0.0000
C	0.3226	1.1084	0.0000
S	2.9441	-0.1507	0.0000
H	0.6803	-2.0520	0.0000
H	0.5871	2.1746	0.0000
Cl	-2.3112	-1.7283	0.0000
**Cl-L6 — (2-Cl-4MC-*N3*H)$_2$ disulfide**			
N	0.3913	0.6632	0.5223
C	1.1739	-0.2173	1.1248
N	2.2146	-0.6283	0.3424
C	2.0967	0.0555	-0.8746
C	0.9516	0.8472	-0.7143
S	3.1602	-0.1903	-2.2264
H	0.5176	1.5310	-1.4555
H	2.9497	-1.2958	0.5872
Cl	0.9645	-0.8212	2.7260
N	7.0019	-0.7327	1.0351

Table S13 (continued)

Atom	x	y	z
C	7.4153	-1.3016	-0.0845
N	6.7915	-0.8071	-1.1951
C	5.8892	0.1663	-0.7520
C	6.0545	0.1779	0.6397
S	4.8863	1.1239	-1.8060
H	5.5342	0.8257	1.3577
H	6.9444	-1.0832	-2.1676
Cl	8.6045	-2.5448	-0.2033
**Cl-C2 — $Cu(I)(2\text{-}Cl-4\,MC-N_3H)_3^+$**			
Cu	0.5769	-0.6932	-0.7122
N	4.8058	1.3691	0.8014
C	5.6801	1.1016	-0.1869
N	5.0046	0.4135	-1.1364
C	3.6519	0.2195	-0.7607
C	3.5531	0.8482	0.4885
S	2.5597	-0.6387	-1.7719
H	5.4030	0.0686	-2.0149
H	2.6791	0.9466	1.1433
H	5.0544	1.8734	1.6564
Cl	7.3220	1.5448	-0.2256
N	-2.4324	2.8276	-0.2784
C	-2.1838	3.4140	0.9085
N	-1.3244	2.6067	1.5755
C	-0.9997	1.4665	0.8027

Table S13 (continued)

Atom	x	y	z
C	-1.7281	1.6329	-0.3821
S	0.0815	0.2443	1.3590
H	-0.9472	2.7894	2.5099
H	-1.7789	0.9721	-1.2580
H	-3.0399	3.2316	-0.9969
Cl	-2.8048	4.9008	1.4490
N	-3.2925	-3.3125	0.7750
C	-3.7682	-3.8942	-0.3426
N	-3.0702	-3.3812	-1.3830
C	-2.1088	-2.4401	-0.9357
C	-2.2761	-2.4174	0.4553
S	-1.0548	-1.6324	-2.0297
H	-3.1927	-3.6429	-2.3659
H	-1.7294	-1.8325	1.2050
H	-3.6353	-3.5271	1.7153
Cl	-5.0047	-5.0590	-0.4206
**Cl-D4 — *cis*-Cu(II)(2-Cl−4 MC^{-})$_2$ (*N3*-binding)**			
Cu	-0.0674	0.8100	-0.1489
N	4.7420	1.7597	-2.1817
C	3.8577	2.7016	-1.9040
N	2.6995	2.1998	-1.3606
C	2.8506	0.8169	-1.2833
C	4.1428	0.5877	-1.8077
S	1.6983	-0.3250	-0.6740

Table S13 (continued)

Atom	x	y	z
H	1.8735	2.7275	-1.0669
H	4.6407	-0.3844	-1.9168
Cl	4.0585	4.3901	-2.1657
N	-3.5624	-0.9451	2.4726
C	-4.5736	-0.2003	2.0648
N	-4.1755	0.8487	1.2727
C	-2.7883	0.7673	1.1547
C	-2.4498	-0.3643	1.9240
S	-1.8719	1.9438	0.2535
H	-4.7755	1.5484	0.8306
H	-1.4390	-0.7558	2.1025
Cl	-6.2363	-0.4556	2.4343
**NO2-L2 — 2-NO_2-4MC thione (*N3*–H, C4=S)**			
N	-0.9452	0.6430	0.0000
C	-0.9416	-0.7091	0.0000
N	0.3544	-1.1157	0.0000
C	1.2387	-0.0007	0.0000
C	0.3346	1.1130	0.0000
S	2.9071	-0.0560	0.0000
H	0.6452	-2.0975	0.0000
H	0.5751	2.1802	0.0000
H	-1.8310	1.1634	0.0000
N	-2.1081	-1.5011	0.0000
O	-1.9406	-2.7354	0.0000

Table S13 (continued)

Atom	x	y	z
O	-3.1984	-0.8873	0.0000
**NO2-L4 — 2-NO_2-4MC$^-$ anion (*N3*–H, *N1*$^-$)**			
N	-1.0002	0.7212	0.0000
C	-0.9711	-0.6183	0.0000
N	0.3253	-1.1026	0.0000
C	1.2093	-0.0257	0.0000
C	0.2995	1.0940	0.0000
S	2.9056	-0.1276	0.0000
H	0.5741	-2.0922	0.0000
H	0.6074	2.1499	0.0000
N	-2.0725	-1.4981	0.0000
O	-1.7981	-2.7359	0.0000
O	-3.2362	-1.0416	0.0000
**NO2-L6 — (2-NO_2-4MC-*N3*H)$_2$ disulfide**			
N	0.3694	0.7652	0.4987
C	1.0855	-0.1609	1.1189
N	2.1251	-0.6357	0.3773
C	2.0816	0.0426	-0.8325
C	0.9726	0.9065	-0.7142
S	3.1658	-0.2600	-2.1685
H	0.6088	1.6106	-1.4741
H	2.7799	-1.3631	0.6821
N	0.8533	-0.6849	2.4637
O	1.6605	-1.5539	2.8340

Table S13 (continued)

Atom	x	y	z
O	-0.0928	-0.2382	3.0998
N	7.1426	-0.6457	0.9716
C	7.4970	-1.2349	-0.1597
N	6.8038	-0.7936	-1.2469
C	5.9138	0.1619	-0.7808
C	6.1586	0.2251	0.6077
S	4.8389	1.0930	-1.7983
H	5.6597	0.8828	1.3321
H	6.9401	-1.1254	-2.2070
N	8.5170	-2.2706	-0.3191
O	8.6709	-2.6798	-1.4821
O	9.1218	-2.6406	0.6790
**NO2-C2 — Cu(I)(2-NO_2−4 MC−N_3H)$_3^+$**			
Cu	0.1218	-0.5479	-0.8917
N	4.4929	0.7773	0.5153
C	5.2106	0.8761	-0.6196
N	4.3510	0.8029	-1.6630
C	3.0339	0.6487	-1.1987
C	3.1637	0.6375	0.2125
S	1.6780	0.5095	-2.2359
H	4.6487	0.8386	-2.6453
H	2.3782	0.5354	0.9707
H	4.9432	0.8050	1.4398
N	6.6316	1.0251	-0.6793

Table S13 (continued)

Atom	x	y	z
O	7.1197	1.0966	-1.8137
O	7.2151	1.0681	0.4140
N	-1.1990	3.9549	-0.1150
C	-1.9472	3.9159	1.0046
N	-1.8178	2.6818	1.5472
C	-0.9642	1.8943	0.7599
C	-0.5823	2.7456	-0.3039
S	-0.5525	0.2672	1.1343
H	-2.2943	2.3940	2.4098
H	0.0827	2.5229	-1.1496
H	-1.1476	4.7994	-0.7006
N	-2.7499	4.9899	1.5005
O	-3.3866	4.7516	2.5350
O	-2.7166	6.0386	0.8410
N	-3.4013	-3.7009	0.4948
C	-3.3613	-4.6824	-0.4256
N	-2.3984	-4.3698	-1.3238
C	-1.7893	-3.1507	-0.9834
C	-2.4643	-2.7464	0.1967
S	-0.5294	-2.4516	-1.9046
H	-2.1649	-4.9571	-2.1330
H	-2.3095	-1.8429	0.7986
H	-4.0629	-3.7286	1.2818
N	-4.1993	-5.8415	-0.4253

Table S13 (continued)

Atom	x	y	z
O	-4.0203	-6.6369	-1.3549
O	-5.0122	-5.9201	0.5073
**NO2-D4 — *cis*-Cu(II)(2-NO_2−4 MC^-)$_2$ (*N3*-binding)**			
Cu	-0.2727	-1.6409	-0.3790
N	1.7433	2.4014	-1.8690
C	2.8788	2.3767	-1.1856
N	3.0606	1.2145	-0.4818
C	1.9578	0.4167	-0.7301
C	1.1567	1.2079	-1.6003
S	1.8213	-1.1941	-0.0816
H	3.8642	1.0226	0.1251
H	0.1838	0.9199	-2.0219
N	3.8821	3.4237	-1.1323
O	4.8424	3.1903	-0.3744
O	3.7168	4.4287	-1.8174
N	-3.7072	0.3059	2.3540
C	-3.2338	-0.0911	1.1562
N	-3.1718	0.9282	0.2400
C	-3.6371	2.0836	0.8620
C	-3.9526	1.6091	2.2025
S	-3.7495	3.6057	0.1742
H	-2.8514	0.8483	-0.7280
H	-4.3302	2.2382	3.0204
N	-2.7139	-1.3564	0.8891

Table S13 (continued)

Atom	x	y	z
O	-2.1382	-1.4341	-0.2995
O	-2.7617	-2.2935	1.6774

References

(S1) Hansch, C.; Leo, A.; Taft, R. W. A Survey of Hammett Substituent Constants and Resonance and Field Parameters. *Chemical Reviews* **1991**, *91*, 165–195.
